# Architecture‐Interface Co‐Design of g‐C_3_N_4_ Driven Dry Thick Cathodes Enabling Fast Li‐Ion Transport

**DOI:** 10.1002/exp2.70207

**Published:** 2026-08-11

**Authors:** Hye Ji Eun, Jinkyu Park, Garam Lee, Young Chul Song, Mihye Wu, Hyojun Choi, Myeong Hwan Lee, Eunki Kim, Young‐Soo Kim, Jiyoung Park, Sang‐Young Lee, Jungdon Suk, San Moon

**Affiliations:** ^1^ Department of Advanced Battery Research Center, Korea Research Institute of Chemical Technology (KRICT) Daejeon Republic of Korea; ^2^ Department of Chemical and Biomolecular Engineering Yonsei University Seoul Republic of Korea; ^3^ Department of Materials Science and Engineering Korea University Seoul Republic of Korea; ^4^ School of International Engineering and Science Jeonbuk National University Jeonju‐si Jeonbuk State Republic of Korea; ^5^ Department of Materials Science and Engineering, College of Engineering Seoul National University Seoul Republic of Korea; ^6^ Department of Energy Science Sungkyunkwan University Susan Republic of Korea; ^7^ Thermo Fisher Scientific Korea Ltd Seoul Republic of Korea

**Keywords:** cathode additive, dry thick‐film electrode, graphitic carbon nitride, high‐rate performance, lithium‐ion battery, pore structure engineering

## Abstract

Overcoming ionic transport limitations in thick‐film electrodes is a central challenge for next‐generation high‐energy‐density lithium‐ion batteries. Here, we report an architecture‐interface co‐design strategy for solvent‐free dry thick cathodes, introducing nanostructured graphitic carbon nitride (g‐C_3_N_4_) as a multifunctional ion‐transport promoter, representing the first demonstration of its application as a cathode additive. Mechanistic studies reveal that g‐C_3_N_4_ operates via a dual mechanism, simultaneously enhancing electrolyte wettability and accelerating Li^+^ transport kinetics through transient Li‐N coordination that effectively lowers the desolvation energy barrier. High‐resolution 3D X‐ray nanotomography and pore network modeling quantitatively map the ionic‐mechanical trade‐off arising from compressive spring‐back in the dry process, directly guiding the optimization of electrode architecture. The resulting single‐layer dry electrode (∼21.5 mg cm^−2^, ∼68 µm thick) delivers a 165.9% capacity increase at 3C and a 2.85‐fold enhancement in power density, while achieving markedly improved cyclic stability with 81.3% capacity retention after 600 cycles. Furthermore, a functionally graded dual‐layer design (∼42.2 mg cm^−2^, ∼113 µm thick) yields a 62.5% capacity gain at 1C. This work establishes a generalizable, scalable, and sustainable pathway for engineering high‐performance dense electrodes.

## Graphical abstract

To break the energy–power trade‐off in dry‐processed thick cathodes, we use g‐C_3_N_4_ as a multifunctional additive. In content‐optimized single‐layer electrodes (∼21.5 mg cm^−2^), transient Li–N coordination lowers Li^+^ desolvation energy and improves wetting; together, these effects raise 3C capacity by 165.9% and power density by 2.85×. A microstructure‐informed dual‐layer architecture (∼42.2 mg cm^−2^) boosts 1C capacity by 62.5%.

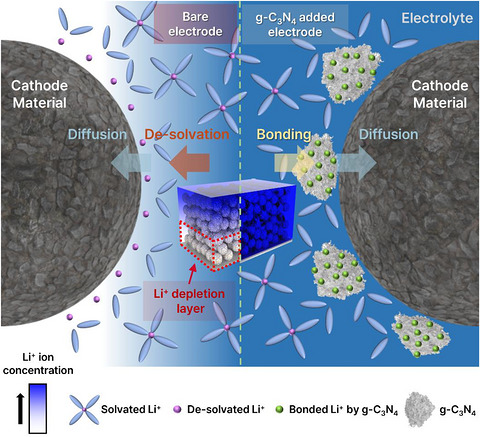

## 1 Introduction

In the modern era, the paramount challenge is not only in generating energy but also in its efficient storage in portable forms. This capability is indispensable given society's growing reliance on portable electronic devices, electric vehicles (EVs), and decentralized energy systems. Central to this transition is the energy density of secondary batteries, particularly lithium‐ion batteries (LiBs), which fundamentally governs the performance and range of these technologies. To enhance the energy density of LIBs, researchers have pursued two principal strategies. The first focuses on increasing the intrinsic capacity of the active materials. Significant progress has been achieved in developing Ni‐rich ternary cathode materials [1, 2, 3, 4, 5, 6, 7], advancing understanding of O_2_ redox mechanisms to overcome capacity barriers [8, 9, 10, 11], and innovating advanced anode materials such as high‐capacity silicon and Li metal [12, 13, 14, 15, 16]. The second strategy is to increase cell‐level energy density through thick‐film electrodes with high active‐material loading and electrode density [17, 18, 19, 20]. Conventional multi‐stacked cells require numerous current collectors and separators, which lower overall energy density [21, 22, 23, 24]. Thus, reducing these non‐active components through thick‐film electrodes is an effective route to higher energy density. However, increasing electrode loading and thickness also raises ionic and electronic resistance and aggravates nonuniform Li^+^ distribution, ultimately compromising battery performance [17, 19, 25, 26, 27, 28, 29, 30, 31, 32, 33, 34].

The key challenge in thick‐film electrode design is managing ionic and electronic transport across powder‐based electrode architectures. Previous studies on thick‐film electrodes have therefore focused on reducing both ionic and electronic resistances [35, 36, 37]. Electronic transport has mainly been improved by incorporating one‐dimensional conductive materials such as carbon nanotubes (CNTs) or carbon nanofibers (CNFs), which form vertical conduction pathways within thick‐film electrodes [17, 37, 38, 39]. In parallel, ionic conductivity must also be optimized by lowering transport tortuosity and improving electrolyte impregnation in the deeper electrode regions. The microstructural characteristics of thick‐film electrodes—including porosity, tortuosity, and pore connectivity—play critical roles in determining ionic transport efficiency through the powder network. Recent studies on architecture engineering of thick electrodes have similarly emphasized that electrolyte accessibility, tortuosity, and transport heterogeneity across the electrode thickness collectively govern the high‐rate performance of practical high‐energy electrodes [40]. Notably, while electronic conductivity is secured by the pre‐dispersed conductive additives, ionic conduction depends solely on the impregnated electrolyte in electrode. As electrode thickness increases, electrolyte impregnation becomes more challenging and the Li^+^ migration distance extends, resulting in a progressively larger contribution of ionic resistance relative to electronic resistance as the primary performance‐limiting factor [30, 31, 35, 36, 41].

These transport limitations are further compounded by electrode manufacturing. In conventional wet processing, solvent evaporation during drying creates compositional gradients across the electrode thickness, thereby impairing both electronic and ionic conduction. In wet‐processed thick‐film electrodes, solvent evaporation (such as NMP, water, etc.) induces upward binder migration, resulting in compositional nonuniformity [42, 43, 44, 45]. Furthermore, thicker electrodes necessitate higher drying temperatures, which exacerbate binder migration [43, 45, 46]. This nonuniform distribution impedes electronic conductivity and reduces ionic conductivity in binder‐rich upper regions, where insufficient binder undermines the mechanical cohesion with the current collector [29, 45, 46].

Dry electrode fabrication is an attractive alternative because it can avoid many limitations of wet processing. In this method, electrode components (active material, binder, and conductive carbon) are blended in a dry state and formed into a cohesive dough using a fibrous binder before being cast into electrodes [29, 43, 44, 45]. This approach mitigates binder migration, lowers process cost, and improves spatial efficiency by eliminating solvent‐drying and recovery steps [43, 45]. As a result, dry electrode fabrication has attracted strong industrial interest, whereas academic investigation remains comparatively limited. However, dry processing does not resolve the intrinsic issue of increased ionic resistance that accompanies greater electrode thickness. Although introducing artificial porosity into thick‐film electrodes is the most direct way to improve Li^+^ conductivity in thick‐film electrodes [47, 48, 49], it inevitably lowers the active‐material fraction and thus reduces overall energy density. Therefore, a key unresolved challenge is to improve Li^+^ transport in dense dry thick electrodes without relying on deliberate pore creation.

In the broader context of fast‐charging/discharging electrochemical energy storage, overcoming such intrinsic ion transport limitations has driven significant innovations in interface modulation and structural engineering. For instance, recent breakthroughs in advanced carbon architectures have demonstrated that modulating van der Waals forces and expanding interlayer spacing in graphene allotropes can drastically lower the energy barrier for ion migration, enabling extreme high‐rate tolerance [50]. Similarly, the rational design of defect‐rich carbon interfaces and pseudocapacitive heterostructures has been shown to facilitate rapid ion flux, successfully realizing 6‐min fast‐charging in practical Ampere‐hour‐scale hybrid capacitors [51]. Beyond structural engineering, tailored interface chemistry has also proven to be a powerful lever for regulating Li^+^ transfer kinetics; for example, SEI reconstruction with SrI_2_ has been shown to create a highly Li^+^‐conductive interphase that suppresses dead Li accumulation in anode‐free batteries [52], and selective ion‐conductive surface coatings have been demonstrated to facilitate fast Li^+^ diffusion while blocking parasitic reactions at the anode–electrolyte interface [53]. Likewise, recent interfacial engineering studies have shown that fast‐charging performance can be markedly improved by homogenizing local ion flux and accelerating interfacial Li^+^ transport through rationally designed lithiophilic heterostructures [54]. These pioneering studies underscore a universal principle: engineering the local coordination environment and optimizing the electrode–electrolyte interface are paramount for accelerating ion transport kinetics under high‐rate conditions.

The development of multifunctional materials that simultaneously address multiple performance‐limiting factors represents a central theme in next‐generation battery research, as highlighted in recent comprehensive reviews on anode‐free lithium batteries [55], electrolyte engineering [56], and data‐driven battery material design [57]. Inspired by these interfacial design principles, we introduce, for the first time, readily accessible graphitic carbon nitride (g‐C_3_N_4_) powder as a multifunctional cathode additive. Although g‐C_3_N_4_ has been explored primarily in anode‐side systems, it offers high surface area, inherent porosity, strong electrolyte wettability, and abundant lithiophilic nitrogen sites [58, 59, 60, 61]. We hypothesized that incorporating g‐C_3_N_4_ into dry thick electrodes could simultaneously improve electrolyte uptake and promote interfacial Li^+^ transfer through transient Li^+^–N coordination, thereby enhancing Li^+^ transport without the energy‐density penalty associated with artificial porosity [58, 59, 62]. On this basis, the present study examines whether g‐C_3_N_4_ can serve as a practical ion‐transport promoter in dry thick cathodes and clarifies how interfacial chemistry and electrode architecture together determine high‐rate performance.

## 2 Results and Discussion

### 2.1 g‐C_3_N_4_‐Enhanced Dry Thick‐Film Electrode Fabrication

The fundamental objective of this study is to improve the rapid discharge performance of thick‐film electrodes. However, fabricating thick‐film electrodes by a wet process remains challenging. Even when successfully produced, the polyvinylidene fluoride (PVDF) binder used in the wet process forms surface contact with the active particles, which restricts ion accessibility and limits active‐material utilization. In contrast, we adopted a dry electrode fabrication method employing a polytetrafluoroethylene (PTFE) binder. In the dry process, the PTFE binder is fibrillated and forms line contact with the active material. This configuration expands the electrochemically active interface (Figure S1a), providing a more favorable basis for high‐rate performance [46, 47, 48].

**FIGURE 1:**
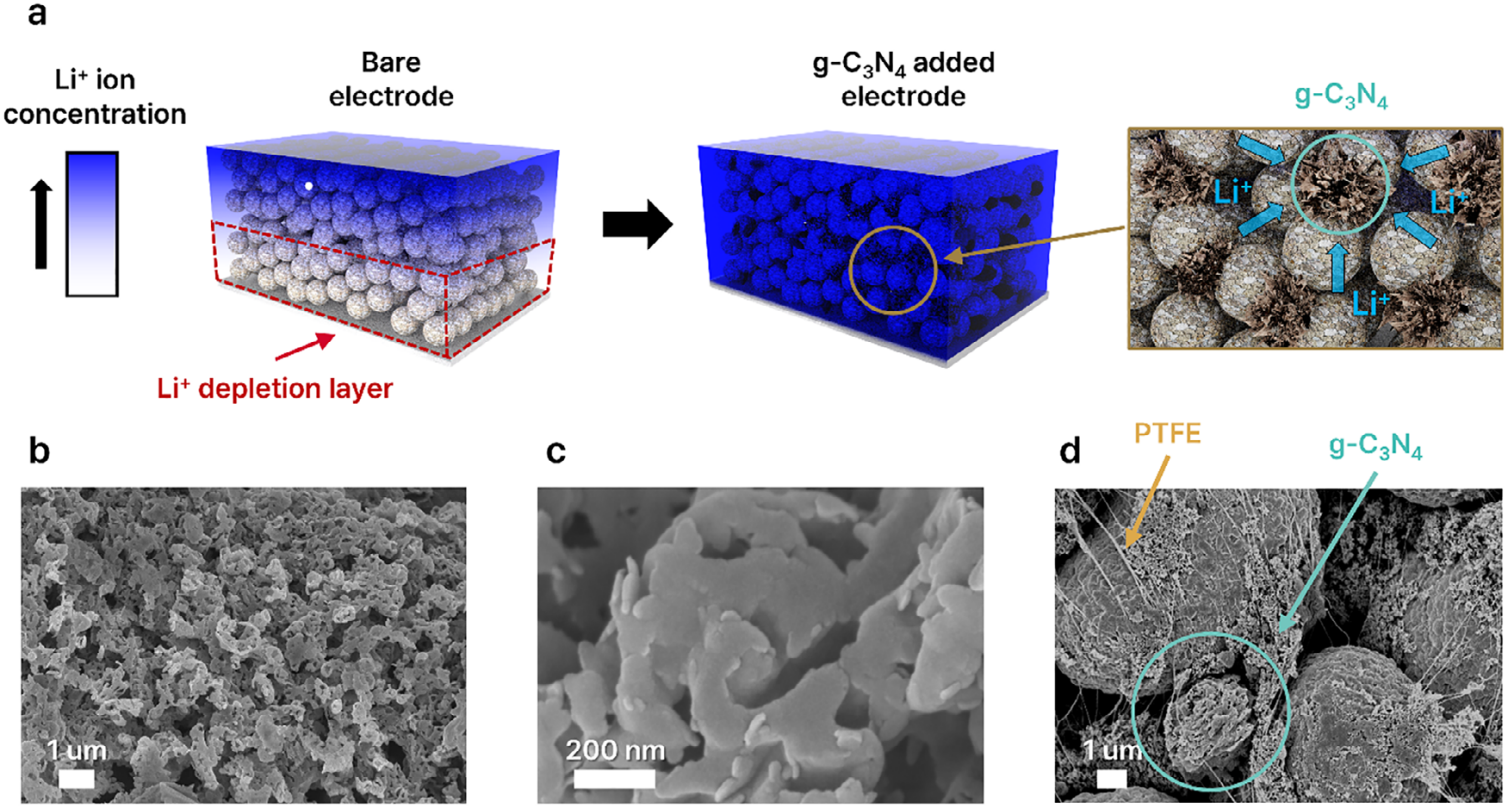
(a) Schematic illustration demonstrating the Li‐ion concentration profiles within thick‐film electrodes fabricated via the dry method during rapid discharge, highlighting the presence of a Li^+^ depletion layer in the bare electrode, and its mitigation through the introduction of graphitic carbon nitride (g‐C_3_N_4_) additives. Insets illustrate the enhanced Li^+^ accessibility facilitated by g‐C_3_N_4_. (b) Low‐magnification and (c) high‐magnification field‐emission scanning electron microscopy (FE‐SEM) images of pristine g‐C_3_N_4_ additive. (d) FE‐SEM image showing the integration of g‐C_3_N_4_ within the electrode structure.

Building on this improved electrode architecture, we then addressed the challenge of enhancing Li^+^ utilization without sacrificing energy density of thick‐film electrode. Although increasing porosity can facilitate Li^+^ conduction, it reduces the active‐material fraction. Conversely, densification shortens the Li^+^ pathway but raises tortuosity and undermines the intended gain [42, 43, 44]. To overcome these opposing constraints, we incorporated graphitic carbon nitride (g‐C_3_N_4_) into the dry thick‐film electrode. We anticipated that the enhanced wettability and lithiophilicity of electrode by g‐C_3_N_4_ would increase the amount of available Li^+^ within the electrode and facilitate Li^+^ supply [58, 59, 60, 61], thereby improving Li^+^ utilization under rapid‐discharge conditions while maintaining high energy density. The Li^+^ concentration profile during rapid discharge is illustrated in Figure 1a. During discharge, Li^+^ diffuses from the electrolyte into the cathode's active material. The upper portion of a thick‐film cathode, directly exposed to bulk electrolyte, can easily replenish consumed Li^+^ at high discharge rates. In contrast, the lower portion must rely solely on electrolyte impregnated in its pores, providing fewer Li^+^ ions. Under slow discharge, there is enough time to restore Li^+^ in the lower region, but under rapid discharge the replenishment rate becomes insufficient. As a result, the upper region undergoes excessive reaction, whereas the lower region remains underutilized, accelerating localized degradation and lowering overall performance [29, 36].

**FIGURE 2:**
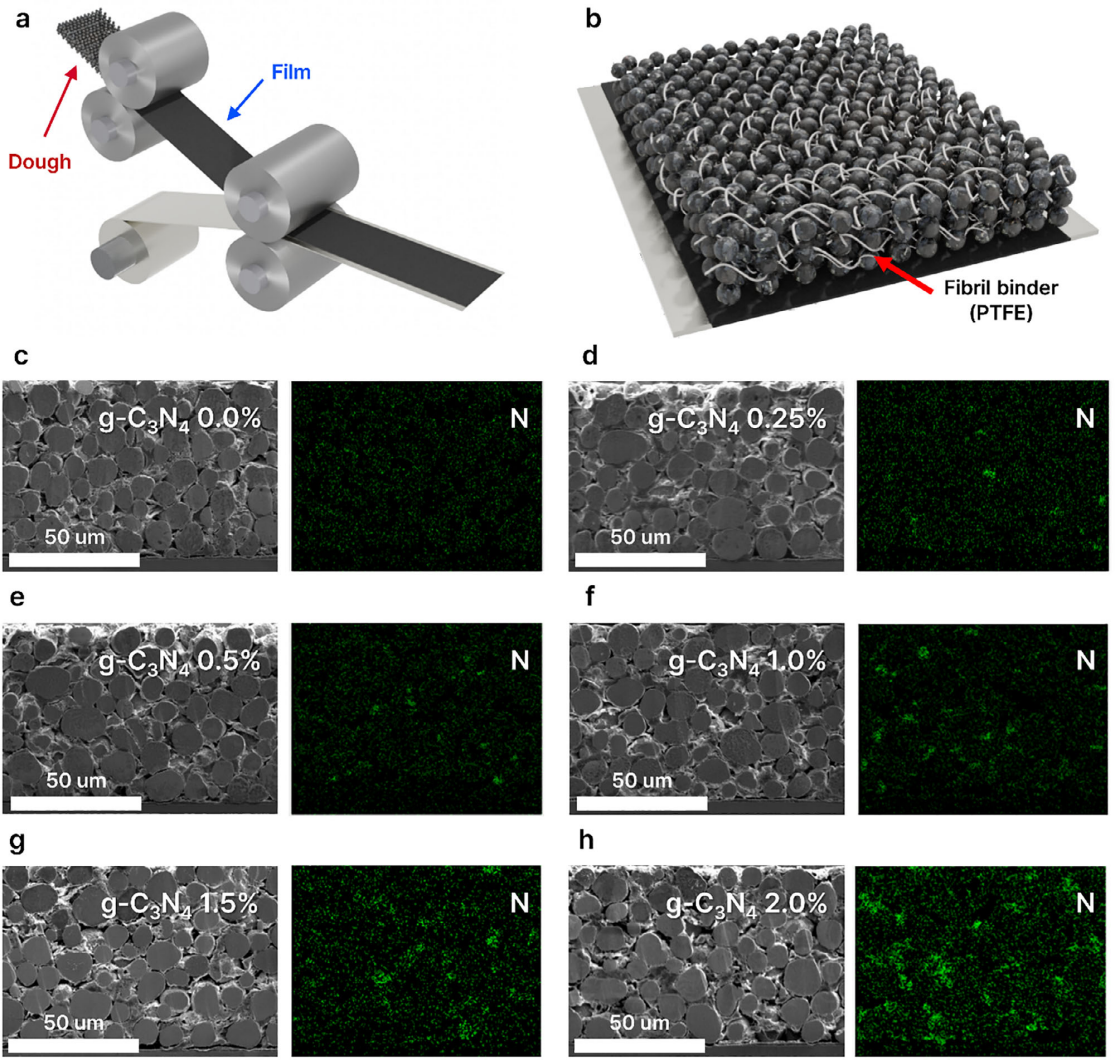
(a) Schematic illustration of the fiberization of polytetrafluoroethylene (PTFE), which is used as a dry electrode binder, through the shear stress transmitted to the electrode via a pressing machine. (b) Illustration of the fibrillized PTFE. (c)–(h) Field‐emission scanning electron microscopy and energy dispersive X‐ray spectroscopy analyses of the cross‐sections of thick‐film electrodes containing (c) 0.0%, (d) 0.25%, (e) 0.5%, (f) 1.0%, (g) 1.5%, and (h) 2.0% g‐C_3_N_4_. A uniform distribution of g‐C_3_N_4_ can be observed within the electrodes.

Nanoporous g‐C_3_N_4_ with a particle size of 500 nm fabricated using a straightforward synthetic method (Figure 1b,c) mixed with the cathode active material, conductive substance, and PTFE binder to form a cohesive dough, and then rolled to generate the desired film. The resulting film (Figure 1d) was prepared as an electrode by integrating it with a current collector using a lamination procedure. The dry electrode fabrication process is illustrated in Figure 2a. Figure 2b shows a 3D rendered image of an electrode fabricated via the dry electrode process. As seen in the SEM image in Figure 1d, the Fibrillated PTFE binder effectively secures all electrode components, preserving the electrode's overall structure. Details of the preparation process for the g‐C_3_N_4_‐incorporated dry thick‐film electrodes are provided in Section 4 and Figure S1b–e. Figure 2c–h presents cross‐sectional views of the prepared electrodes, as observed via FE‐SEM and energy dispersive X‐ray spectroscopy (EDS) analysis. EDS confirmed an increase in N species within the electrode as the g‐C_3_N_4_ content of the electrode increased; moreover, the N was distributed uniformly over the electrode. This finding indicates the uniform dispersion of the additive throughout the dry thick‐film electrode. The resultant electrodes exhibited a loading capacity of approximately 21.5 mg/cm^2^, thicknesses ranging from 68 to 69 µm, and an electrode density of ∼3.0 g/cc, as summarized in Table S1.

Previous research has documented the ability of g‐C_3_N_4_ to enhance the wettability of the electrolyte, a factor closely linked to the amount of liquid electrolyte absorbed by the electrode [59, 61]. To validate this enhancement, we measured the contact angle of the electrolyte on both the g‐C_3_N_4_‐integrated electrodes and a control electrode. The electrolyte used was identical to that employed in the electrochemical evaluations. The results demonstrated that the electrolyte wettability increased as the amount of g‐C_3_N_4_ incorporated into the electrodes increased (Figure S2). Such additive‐enabled improvement in thick‐electrode behavior is consistent with recent reports showing that multifunctional additives can simultaneously regulate ion/electron transport and structural characteristics in ultrathick electrode systems [63]. The improved wettability suggests a stronger tendency for electrolyte uptake within the electrode, which is expected to increase Li^+^ accessibility around the cathode active materials, although the penetration depth itself was not directly visualized in this study. In essence, enhancements in electrolyte absorption owing to the incorporation of various amounts of g‐C_3_N_4_ into the electrodes can be considered advantageous, particularly in terms of the quantity of Li^+^ available during the initial rapid‐discharge phase. However, the replenishment of the depleted Li^+^ following the initial rapid discharge may be influenced by factors beyond the wettability characteristics attributed to g‐C_3_N_4_.

### 2.2 Li–N Bond Formation and Li^+^ Conductivity in g‐C_3_N_4_‐Enhanced Dry Thick‐Film Electrodes

**FIGURE 3:**
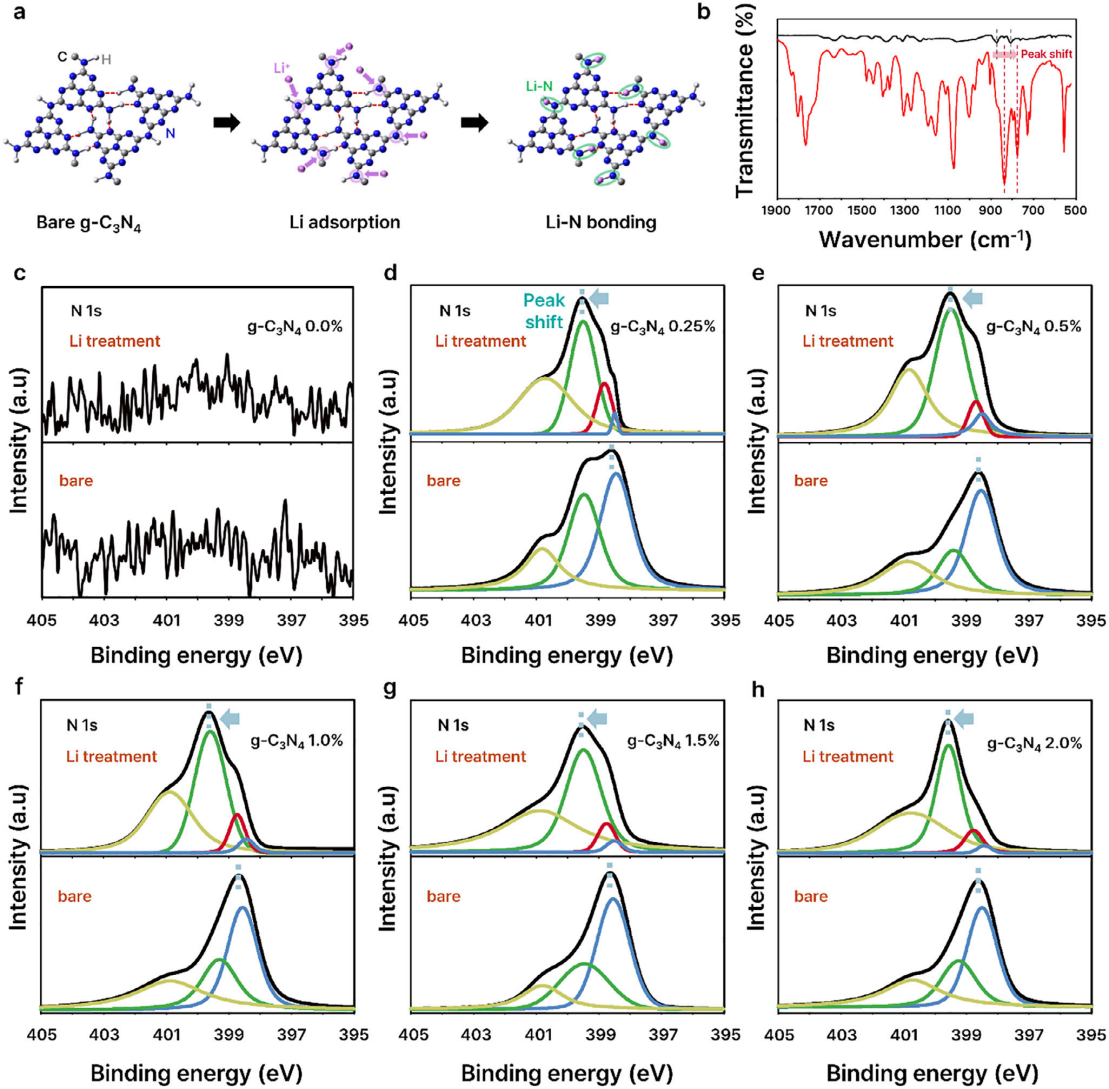
(a) Schematic illustration of temporary Li–N bond formation in graphitic carbon nitride (g‐C_3_N_4_). (b) Fourier‐transform infrared spectrum of the 750–850 cm^−1^ region of a g‐C_3_N_4_ sheet before and after impregnation with the electrolyte (1.15 m LiPF_6_ in EC:EMC (3:7) + 10% FEC). (c)–(h) N 1s X‐ray photoelectron spectra obtained before and after the electrolyte impregnation of the electrodes with (c) 0.0%, (d) 0.25%, (e) 0.5%, (f) 1.0%, (g) 1.5%, and (h) 2.0% g‐C_3_N_4_.

g‐C_3_N_4_ has been reported to have abundant N species, which exhibit a propensity for Li^+^ interactions [58, 59]. We hypothesized that the dangling lone‐pair electrons of N atoms in g‐C_3_N_4_ would serve as Lewis base sites, effectively drawing in Li^+^, which is characterized as a Lewis acid, through acid–base interactions (Figure 3a). According to this mechanism, the g‐C_3_N_4_ incorporated into the electrode structure initiates a distinctive chemical interaction when the electrode is immersed in a lithim salt‐dissolved electrolyte. The N atoms in g‐C_3_N_4_, which are endowed with lone‐pair electrons, exhibit strong affinity for Li^+^, thereby facilitating Li^+^ desolvation and leading to the establishment of transient Li–N bonds [64, 65, 66]. These bonds are dynamically formed and broken during the Li^+^ transport process, wherein the N atoms temporarily coordinate with solvated Li^+^, lowering the desolvation energy barrier, and subsequently release the desolvated Li^+^ for intercalation into the active material. Semi‐quantitative evidence for this coordination was obtained from the deconvoluted N 1s XPS spectra recorded before and after Li treatment (Table S3). Before Li treatment, the Li‐N peak at 398.7 eV was absent in all g‐C_3_N_4_‐containing electrodes, confirming that this species does not exist in the as‐prepared state. After Li treatment, however, the Li‐N peak emerged clearly, accounting for 8.2%–11.9% of the total N 1s area depending on g‐C_3_N_4_ content. Correspondingly, the Sp^2^ N peak at 398.5 eV, associated with pyridinic nitrogen in the triazine rings, decreased substantially after Li treatment (e.g., from 43.1% to 16.2% at 0.5 wt%), indicating that the lone‐pair electrons of pyridinic nitrogen atoms are partially consumed through Li^+^ coordination. Consistent results were observed in the Li 1s spectra (Table S4), where the Li‐N component at 55.2 eV was completely absent in the electrode without g‐C_3_N_4_ but represented 26.0%–31.2% of the total Li 1s area in all g‐C_3_N_4_‐containing electrodes after Li treatment. Because XPS is inherently surface‐sensitive, these values represent semi‐quantitative descriptors of surface Li‐N coordination rather than the absolute Li^+^ capture capacity of the entire electrode.

The uniform dispersal of g‐C_3_N_4_ across the electrode increases the overall Li^+^ content by introducing additional Li^+^ through Li–N bonding, beyond the Li^+^ naturally provided by the electrolyte. This interaction may not only augment the Li^+^ concentration within the electrode but also help replenish Li^+^ once it is depleted, thereby enhancing the electrochemical performance of the electrode. This aspect is of critical importance during intense discharge periods, where the rapid depletion of Li^+^ within the electrolyte impregnated in the electrode and the subsequent challenge of quickly restoring these ions into the electrode, especially near the current collector in thick‐film electrodes, are pronounced [64, 65, 66].

Fourier‐transform infrared (FTIR) spectroscopy was conducted on an sheets composed exclusively of g‐C_3_N_4_ and PVDF before and after immersion in a Li‐salt electrolyte, followed by washing (hereafter referred to as Li treatment) (Figure S3). The results revealed a discernible peak shift in the 750–850 cm^−1^ spectral range (Figure 3b). This spectral region corresponds to the out‐of‐plane bending vibrations of C–N–C linkages in the heptazine ring units of g‐C_3_N_4_ [67, 68]. The observed shift toward lower wavenumbers is consistent with the coordination of Li^+^ to the pyridinic N atoms, which elongates the local C–N bonds through electron density redistribution, thereby reducing the bond force constant and lowering the vibrational frequency [69]. This spectral deviation is attributed to the perturbation of the intrinsic bond vibrations by the extraneous Li^+^, supporting the hypothesis of Li–N bond formation within g‐C_3_N_4_ upon its immersion in the electrolyte solution [59, 70]. These experimental findings agree with our initial expectation and underscore the proposed chemical role of the additive.

FTIR analysis substantiated the formation of Li–N bonds within the g‐C_3_N_4_‐electrolyte system. To determine whether this phenomenon extends to our thick‐film electrodes, we examined electrodes with different g‐C_3_N_4_ contents using X‐ray photoelectron spectroscopy (XPS) before and after Li treatment (Figure 3c–h and Figure S4). Figure S4 depicts the Li 1s XPS profiles of the thick‐film electrodes with different g‐C_3_N_4_ compositions after Li treatment. The XPS profile of the electrode without g‐C_3_N_4_ showed peaks at 55.0 and 55.8–56.0 eV (Table S2), corresponding to LiOH/Li_2_CO_3_ (residual Li in the active materials) and LiF (a byproduct of the electrolyte–active material interactions), respectively [71, 72, 73]. LiF can materialize even in the absence of an electrochemical reaction owing to the exposure of the highly Ni‐layered oxide to the LiPF_6_‐desolved electrolyte [74, 75]. After Li treatment, the g‐C_3_N_4_‐integrated electrodes revealed a peak at 55.2 eV, characteristic of Li_3_N, confirming the formation of Li–N bonds between Li^+^ and the N atoms in g‐C_3_N_4_ [59, 76]. Li–N bonding was further evidenced by a rightward shift in the overall Li 1s XPS profiles of the g‐C_3_N_4_‐integrated electrodes.

Figure 3c–h depicts the N 1s spectra of both the untreated and Li‐treated electrodes. No distinct N 1s peak was observed in the electrode without g‐C_3_N_4_, regardless of its state (untreated or Li treated; Figure 3c), presumably because of the absence of N‐containing bonds in either the cathode active material or binder. By contrast, N 1s peaks were evident in the profiles of the g‐C_3_N_4_‐integrated electrodes. As shown in Figure 3d–h, the untreated electrodes showed three distinct peaks at 398.5, 399.5, and 400.8 eV, corresponding to sp^2^ N atoms in the triazine ring, N atoms bridging N–(C)_3_ structures, and N–H bonds, respectively (Table S2) [62]. After Li treatment, an additional peak at 398.7 eV was observed, indicating the formation of a Li–N bond [59, 77]. The emergence of this peak led to an overall leftward shift in the N 1s spectrum of the g‐C_3_N_4_‐integrated electrodes. These XPS findings align with our earlier FTIR results and further reinforce the evidence for Li–N bond formation after electrolyte infusion, reaffirming the role of g‐C_3_N_4_ in increasing Li^+^ concentration within the electrode and promoting uniform electrochemical reactions.

The increase in Li^+^ concentration owing to g‐C_3_N_4_ integration results from enhanced electrolyte impregnation within the electrode due to improved electrolyte wettability and the incorporation of additional Li^+^ through Li–N bond formation within g‐C_3_N_4_. This augmentation may beneficially delay Li^+^ depletion during rapid discharge. However, as this effect remains limited to the initial stage, further investigation is required to evaluate whether g‐C_3_N_4_ affects electrode performance not only at the initial stage but throughout the entire rapid discharge process.

To delineate the factors affecting the reaction kinetics throughout the discharge process, we measured Li^+^ charge‐transfer resistance (R_ct_) using temperature‐dependent electrochemical impedance spectroscopy (EIS) to estimate the activation energy required for Li^+^ charge transfer. Application of the *R*_ct_ values to the Arrhenius equation will enable the calculation of the activation energy required for Li^+^ diffusion at the interface between the cathode active material and electrolyte [78, 79]. The EIS measurements were conducted from 283.15 to 323.15 K. The data derived from these measurements aligned closely with the straight‐line fits according to the Arrhenius equation:

(1) $$\;\frac{T}{R_{\mathrm{ct}}}=\mathrm{Aexp}\left(-\frac{E_{a}}{\mathrm{RT}}\right)$$

In the Arrhenius equation applied here, 𝐸_𝑎_ represents the activation energy, 𝑇 denotes the absolute temperature, 𝑅 is the universal gas constant, 𝑅_𝑐𝑡_ refers to the resistance to interfacial Li^+^ transfer, and 𝐴 is a pre‐exponential factor.

**FIGURE 4:**
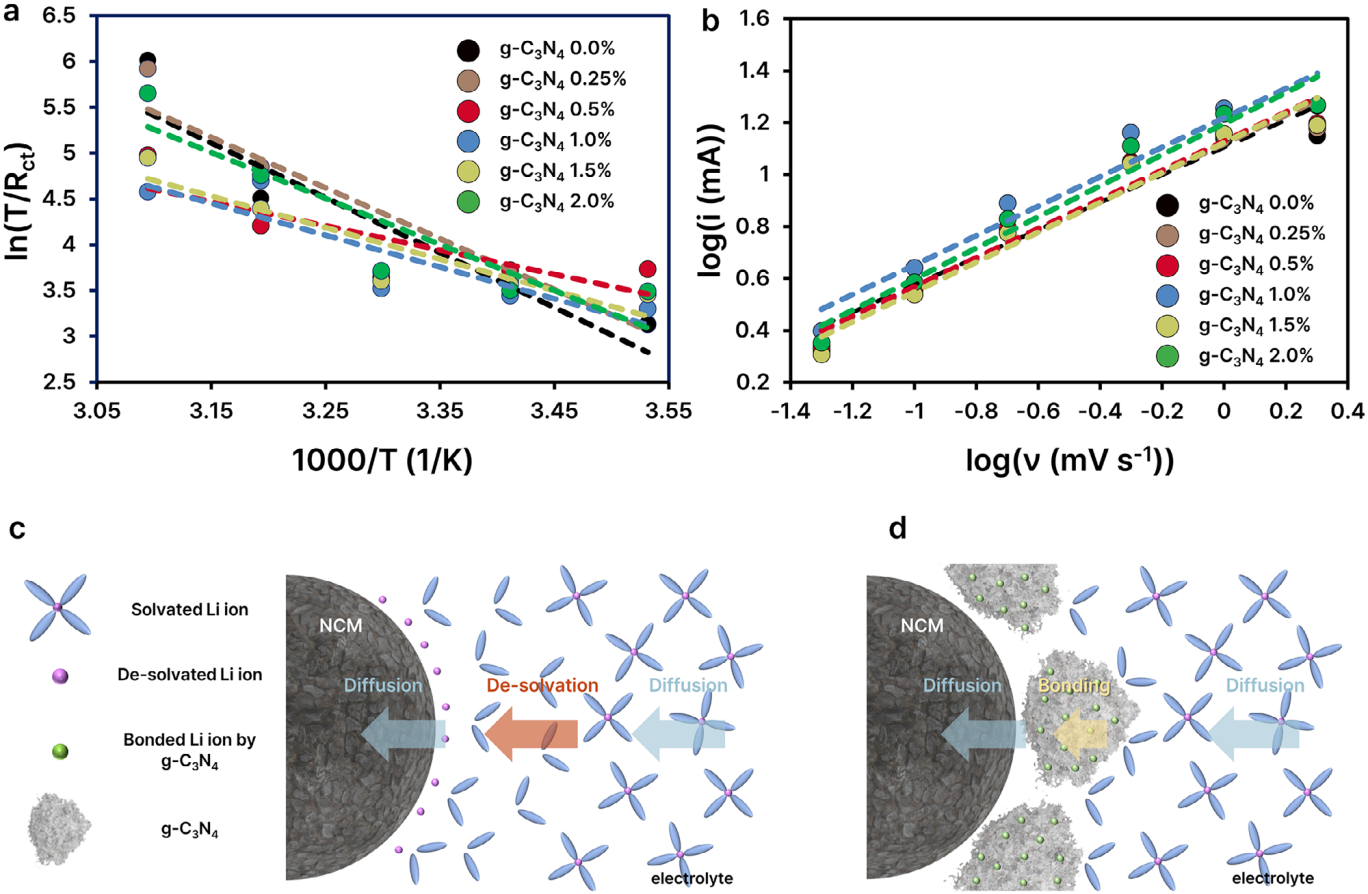
(a) Activation energy of half‐cells composed of electrodes with and without g‐C_3_N_4_ based on the relationship between ln *R*_ct_ and 1/*T*. (b) Capacitance effect in half‐cells composed of electrodes with and without graphitic carbon nitride (g‐C_3_N_4_). (c) and (d) Schematic illustrations of the general reaction steps and energy barriers occurring on the surface of cathode active materials (c) without and (d) with g‐C_3_N_4_. The presence g‐C_3_N_4_ significantly lowers the energy barrier for Li^+^ diffusion, thereby improving the high‐rate performance of the electrode.

Figure 4a depicts the variation in the activation energy of the Li^+^ charge‐transfer process under different g‐C_3_N_4_ concentrations. This property represents the energy barrier that Li^+^ must surmount to traverse the interface between the electrolyte and cathode active materials. The methodologies employed in this experiment and results are detailed in Figure S5 and Table S6. The experiment revealed a reduction in activation energy in g‐C_3_N_4_‐integrated electrodes, highlighting the pivotal role of g‐C_3_N_4_ in enhancing Li^+^ diffusion. However, the activation energy of the Li^+^ charge‐transfer process did not linearly decrease with increasing g‐C_3_N_4_ content (Table S6). Instead, the activation energy decreased as the g‐C_3_N_4_ content increased up to 0.5%, where it reached its minimum value, but further addition of g‐C_3_N_4_ led to an increasing trend in activation energy.

Elucidating the nonlinear correlation between activation energy and g‐C_3_N_4_ content necessitates a comprehensive understanding of the Li^+^ charge‐transfer mechanism at the electrode–electrolyte interface. Li^+^ charge transfer process involves the formation of Li_x_MeO_2_ by intercalation of solvated Li^+^ in the electrolyte to in the active material, completed by electron acceptance from the active material [80]. This encompasses the desolvation of Li^+^ prior to its entry into, and subsequent diffusion through, the cathode‐electrolyte interphase (CEI) [81, 82, 83], involving two critical rate‐limiting steps: (1) the desolvation of Li^+^ and (2) its transport through the CEI. Our FTIR and XPS analyses showed that the incorporation of g‐C_3_N_4_ into the electrode assists in Li^+^ desolvation prior to the electrochemical reaction by the formation of Li–N bonds. Based on these observations, it can be posited that an increase in g‐C_3_N_4_ content within the electrode reduces the energy required for desolvation, consequently lowering the activation energy necessary for Li^+^ charge transfer process. This finding is consistent with broader efforts in which functional groups or polar interfaces are engineered to modify the Li^+^ solvation sheath and reduce the desolvation energy penalty, as demonstrated through quantitative SEI analysis [84], anion‐dipole‐mediated solvation modulation in weakly solvated electrolytes [85], and porous framework design for enhanced Li^+^ transport kinetics [86]. However, an increase in activation energy is observed when the g‐C_3_N_4_ content exceeds 0.5%. This change is attributed to the diffusion of Li^+^ through the CEI, which represents the second rate‐limiting step in Li^+^ charge‐transfer process. This diffusion step involves the acceptance of an electron by Li^+^ to form Li_x_MeO_2_ at the interface between the cathode material and CEI. The cathode‐electrolyte interphase (CEI) generally forms as an ultrathin layer after cell formation, consistent with our electrochemical impedance spectroscopy (EIS) analysis, where the CEI resistance (R_CEI_) appeared negligible and indistinguishable (Figure S5) [87, 88, 89]. Therefore, it can be reasonably inferred that Li^+^ diffusion within the CEI is minimally affected by variations in the electrode's g‐C_3_N_4_ content. Instead, the critical rate‐limiting factor is the acceptance of electrons by Li^+^ at the active material/CEI interface, to form Li_x_MeO_2_ after traversing the CEI.

Previous studies have reported the electrical conductivity of g‐C_3_N_4_ to be approximately 0.9 × 10^−8^ mS cm^−1^, which is considerably lower than the conductivity of the cathode active material used in our experiments (0.0245 × 10^−3^–6.14 mS cm^−1^); thus, g‐C_3_N_4_ exhibits almost insulator‐like properties [90, 91, 92]. Figure S6 illustrates the relationship between the electrical resistance of the electrode and its g‐C_3_N_4_ content. As the g‐C_3_N_4_ content increases, the electrical resistance of the electrode increases. This result implies greater difficulty for the active material in the electrode to accept electrons with increasing g‐C_3_N_4_ concentration. In essence, while the increase in g‐C_3_N_4_ content reduces the energy required for Li^+^ desolvation owing to the early formation of Li–N bonds, which decreases the activation energy barrier, it concurrently increases the electrical resistance of the electrode. Consequently, the rate at which Li^+^ ions combine with electrons after traversing the CEI, a key step in Li^+^ charge‐transfer, is reduced, indicating that activation energy increases when the g‐C_3_N_4_ content exceeds 0.5%.

To elucidate the Li‐storage mechanisms of g‐C_3_N_4_, we fabricated half‐cells with electrodes containing various concentrations of g‐C_3_N_4_ and subjected them to cyclic voltammetry tests. During these tests, the distinct redox peaks observed varied with the increase in sweep rate (ν), correlating with the equation:

(2) $$i\;=\alpha \nu^{b}$$

where *b* describes the charge‐storage mechanism [93].

Typically, the *b*‐value of 0.5 signifies semi‐infinite linear diffusion control of the current, whereas a value of 1 indicates surface control of the current [94]. The *b*‐values were determined by the linear fitting of the data from the half‐cell experiments to Equation (2) (Figure 4b). Details of the experimental methods and data are provided in Figure S7 and Table S7, respectively. The electrodes without g‐C_3_N_4_ had a *b*‐value of 0.5287; and *b*‐value increased following g‐C_3_N_4_ addition, peaking at 0.5978 with 2% g‐C_3_N_4_. This change indicates a transition from a diffusion‐limited process to a more surface‐controlled process within the electrode. This transition in the b‐value provides critical insight into the specific pathway of Li^+^ diffusion facilitated by the additive. It strongly suggests that Li^+^ predominantly migrates along the surface and through the nanoporous channels of g‐C_3_N_4_, rather than intercalating deeply into its interlayer spaces. The abundant nitrogen atoms exposed on the surface of g‐C_3_N_4_ engage in rapid, transient Li‐N coordination, which effectively lowers the desolvation energy barrier. Because this interaction is surface‐dominated and temporary, the desolvated Li^+^ ions can swiftly migrate across the g‐C_3_N_4_ interface via a site‐to‐site hopping mechanism [95] and subsequently intercalate into the adjacent cathode active material, thereby accelerating the overall charge‐transfer kinetics without becoming trapped within the additive's bulk structure.

To further correlate the reduced activation energy (Ea) with the improved fast‐charging performance, we estimated the theoretical enhancement in the charge‐transfer rate using the Arrhenius relationship ($k\propto \exp(-\frac{E_{a}}{\mathrm{RT}})$). The substantial reduction in Ea from 49.8 to 22.1 kJ mol^−1^ upon 0.5% g‐C_3_N_4_ addition (delta 𝐸_𝑎_ = 27.7 kJ mol^−1^) represents a 56% decrease in the energy barrier for interfacial Li^+^ charge transfer. Given the exponential dependence of reaction rate on activation energy in the Arrhenius framework, this reduction translates to a dramatic, non‐linear acceleration of charge‐transfer kinetics at the electrode–electrolyte interface. This kinetic enhancement directly underpins the 165.9% capacity improvement observed at 3C, where the electrode must sustain a Li^+^ flux three times greater than at 1C. Under such demanding conditions, even a modest improvement in interfacial kinetics yields disproportionately large gains in accessible capacity.

Taken together, as depicted in Figure 4c, the diffusion process of Li^+^ in a standard electrode typically involves ion diffusion within the electrolyte, followed by Li^+^ desolvation near the cathode active material and, subsequently, a diffusion reaction through the surface of the cathode. This reaction pathway requires considerable energy for Li^+^ desolvation, which acts as a rate‐limiting step. However, as illustrated in Figure 4d, Li^+^ desolvation partially occurs in electrodes containing g‐C_3_N_4_ and Li–N bonds are transiently formed, substantially reducing the energy barrier for Li^+^ diffusion. Consequently, the overall kinetics of Li^+^ diffusion within the electrode is accelerated.

### 2.3 Electrochemical Performance and Li^+^ Distribution in g‐C_3_N_4_‐Enhanced Dry Thick‐Film Electrodes

**FIGURE 5:**
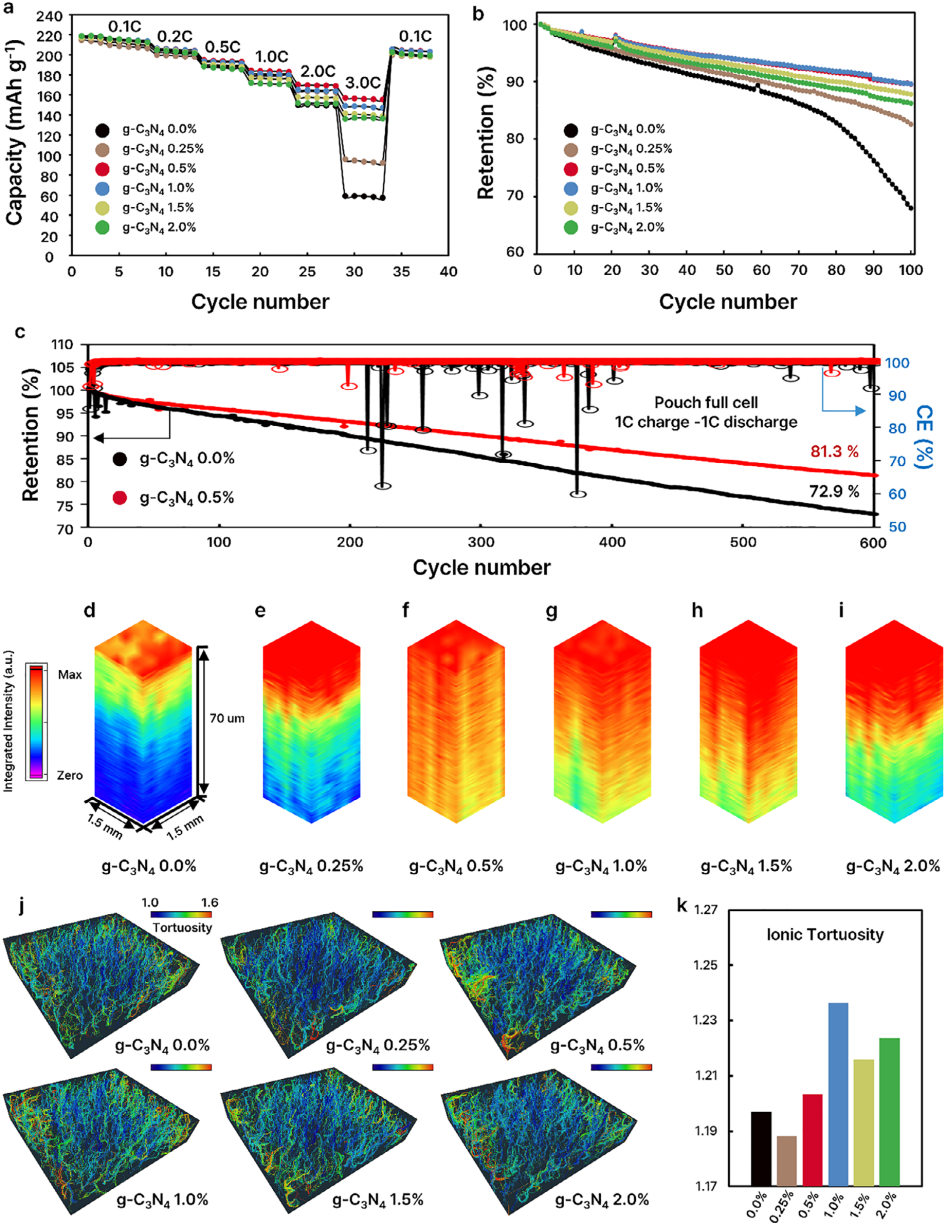
Analysis of the electrochemical performance and laser‐induced breakdown spectroscopy (LIBS) profiles of electrodes with or without graphitic carbon nitride (g‐C_3_N_4_). (a) Rate performance at different discharge rates (0.1C–3.0C), and cycling performance of (b) half‐cells at 1C and (c) full‐cells at 0.5C, with and without g‐C_3_N_4_. (d)–(i) LIBS analysis of the Li mapping images of electrodes containing (d) 0.0%, (e) 0.25%, (f) 0.5%, (g) 1.0%, (h) 1.5%, and (i) 2.0% g‐C_3_N_4_. (j) Tortuosity simulation results based on pore structures reconstructed from 3D X‐ray microscopy images of electrodes using Avizo software. (k) Quantitative tortuosity values derived from the simulations shown in (j).

The electrochemical performance of the thick‐film electrodes was evaluated using coin‐type half‐cells (Figure 5a and Table S1). Details of the cell preparation steps and measurement conditions are provided in Section 4. To assess the discharge characteristics of the electrodes, we set a consistent charge rate of 0.1C and incrementally increased the discharge rate from 0.1C to 3.0C. This assessment aimed to explore the role of g‐C_3_N_4_ during rapid discharge in thick‐film electrodes, particularly when the existing Li^+^ near the active materials is exhausted. Rate testing after cell formation showed comparable capacities at 0.1C and 0.2C across all electrodes, with values of approximately 215 and 205 mAh g^−1^, respectively. At higher discharge rates, however, the electrodes containing g‐C_3_N_4_ showed clear capacity improvements relative to the g‐C_3_N_4_‐free electrode. At 2C, the electrodes containing 0.5%, 1.0%, and 1.5% g‐C_3_N_4_ exhibited capacity increases of approximately 13.3%, 9.9%, and 5.1%, respectively. The difference became much more pronounced at 3C. Relative to the g‐C_3_N_4_‐free electrode, the capacity increased by about 60% with 0.25% g‐C_3_N_4_ and by 165.9% with 0.5% g‐C_3_N_4_, the highest value among the tested compositions. At higher additive contents, the gain gradually declined to 152.5%, 139.0%, and 132.9% for 1.0%, 1.5%, and 2.0% g‐C_3_N_4_, respectively. Although the degree of capacity improvement varied with the amount of g‐C_3_N_4_ added, it is noteworthy that the addition markedly enhanced high‐speed discharge performance. Under 3C discharge conditions, the electrode incorporating 0.5% g‐C_3_N_4_ exhibited both a higher nominal voltage (3.51 V) and increased capacity compared to the electrode without g‐C_3_N_4_ (3.25 V), indicating the potential to develop batteries with up to 2.85 times greater power density (Figure S8 and Table S8). To further distinguish Li–N‐coordination‐assisted kinetics from simple electrolyte‐wettability enhancement, we prepared a high‐temperature‐treated g‐C_3_N_4_ control sample (HT‐g‐C_3_N_4_; Figure S9 and Table S9). HT‐g‐C_3_N_4_ exhibited markedly reduced BET surface area, pore volume, and electrolyte wettability compared with pristine g‐C_3_N_4_, while retaining the Li–N coordination signature after Li treatment. The HT‐g‐C_3_N_4_‐containing electrode still outperformed the additive‐free electrode but did not reach the capacity of the pristine g‐C_3_N_4_ electrode, indicating that Li–N coordination contributes to high‐rate performance beyond simple wettability enhancement, whereas the full benefit of pristine g‐C_3_N_4_ arises from the cooperative action of wettability and exposed Li–N coordination sites. On the other hand, the increased electrical resistance of electrodes with higher g‐C_3_N_4_ content, as discussed previously, may partially explain the observed deterioration in rapid discharge characteristics at concentrations above 0.5%. Nevertheless, it is essential to investigate the structural alterations of electrode induced by the incorporation of a substantial amount of additive, which will be discussed in greater detail subsequently.

The longevity traits of the electrodes mirrored the patterns observed during high‐speed discharge. The electrode without g‐C_3_N_4_ maintained 68.0% of its capacity after over 100 cycles at 1C discharge, whereas the electrode with 0.5% g‐C_3_N_4_ exhibited an enhanced capacity retention of 89.6% (Figure 5b). Furthermore, when assembled into pouch full‐cells for extended cycling evaluation, the electrode containing 0.5% g‐C_3_N_4_ retained 81.3% of its capacity after 600 cycles, whereas the counterpart without g‐C_3_N_4_ showed a lower retention of 72.9% under the same conditions (Figure 5c and Table S10). This result is in line with recent fast‐charging electrode studies showing that kinetically favorable material design can improve not only high‐rate accessibility but also long‐term cycling durability under demanding operating conditions [96]. This improved lifespan is attributed to Li^+^ homogenization within the electrode, which is facilitated by g‐C_3_N_4_. Uneven Li^+^ distribution accelerates degradation of the cathode active material, particularly at the electrode's upper region, due to non‐uniform electrochemical reactions occurring across different electrode sections [27, 97]. Such degradation is accompanied by an increased formation of the rock‐salt phase and crack propagation in high‐Ni cathode materials. These structural changes occur primarily at the upper region due to reaction with excess Li^+^ and substantially reduce the electrode's overall performance. Moreover, cathode material at the lower portion of the thick‐film electrode often remain unreacted owing to inherent challenges in replenishing the depleted Li^+^ during rapid discharge [17], exacerbating reaction inhomogeneity between the upper and lower regions and ultimately impairing the electrode's long‐term lifespan.

To examine the Li^+^ distribution within the electrode under rapid‐discharge conditions, we performed laser‐induced breakdown spectroscopy (LIBS). LIBS determines elemental composition by generating a high‐temperature plasma with a laser pulse and analyzing the resulting optical emission [98, 99]. We scanned the electrode from top to bottom and monitored the Li^+^ signal across the thickness. Figure S10 shows the LIBS profiles of bare electrodes containing 0% and 2.0% g‐C_3_N_4_ before electrochemical testing. In both cases, the active material was fully lithiated, giving a nearly uniform Li^+^ distribution throughout the electrode. We then charged a series of electrodes containing 0%–2.0% g‐C_3_N_4_ at 0.1C and discharged them at 3C. Under this high‐rate condition, the Li^+^ stored in the impregnated electrolyte is rapidly consumed and must be replenished from the bulk electrolyte. As shown in Figure 5d–i, the g‐C_3_N_4_‐free electrode exhibited a Li‐rich top region but a Li‐deficient bottom region, indicating incomplete reaction near the current collector. By contrast, the g‐C_3_N_4_‐containing electrodes showed a much more uniform Li^+^ distribution across the thickness, indicating more uniform utilization of the cathode during rapid discharge. Additionally, similar to the outcomes of the C‐rate assessments, Li^+^ distribution within the electrode was most uniform at 0.5% g‐C_3_N_4_ content, while electrodes with 1.0% and 1.5% g‐C_3_N_4_ showed a slight decrease in distribution uniformity. At 2.0% g‐C_3_N_4_, the consistency of the discharge reaction between the top and bottom of the electrode deteriorated significantly.

The enhancements in thick‐film electrode performance, as evidenced by electrochemical testing and LIBS analysis, can be primarily attributed to two key effects of g‐C_3_N_4_ incorporation:

1. One of the core effects of g‐C_3_N_4_ incorporation is the increase in Li^+^ concentration within the electrode. The enhanced electrolyte wettability induced by its porous nanostructure promotes greater electrolyte uptake, and the formation of Li–N bonds contributes additional Li^+^ to the electrode. As a result, more Li^+^ is available during the initial stage of high‐rate discharge, effectively delaying Li^+^ depletion around the cathode active material.
2. g‐C_3_N_4_ also contributes to the enhancement of Li^+^ diffusion kinetics across the electrode. The formation of Li–N bonds lowers the desolvation energy of Li^+^, and the intrinsic Li^+^ affinity of g‐C_3_N_4_ facilitates more efficient ion transport. This combined effect promotes continuous and rapid Li^+^ migration, supporting the overall discharge process from beginning to end.

### 2.4 Spring‐Back Induced Architectural Rearrangement and Dual‐Layer Design

Based on these advantages, it could be inferred that the high‐rate discharge performance of thick‐film electrode should progressively improve as the g‐C_3_N_4_ content increases. However, the fact that high‐rate discharge performance does not continue to improve with increasing g‐C_3_N_4_ content suggests that excessive incorporation of g‐C_3_N_4_ may introduce adverse effects beyond the previously discussed reduction in electrical conductivity of electrodes. The densities of g‐C_3_N_4_ (1.68 g/cm^3^) and the high‐Ni cathode material (4.53 g/cm^3^) were measured using a gas pycnometer, confirming a substantial difference between the two (Figure S11 and Table S11). Given that all electrode samples were fabricated with similar packing density (∼3.0 g/cc), the excessive addition of g‐C_3_N_4_ may have altered the internal electrode structure in a manner that impedes Li^+^ transport.

To elucidate how such structural alterations induced by excessive g‐C_3_N_4_ incorporation might impede Li^+^ transport during discharge, it is essential to first understand the specific lithium‐ion diffusion pathways within the electrode. Li^+^ insertion into the electrode occurs through four distinct diffusion pathways (Figure S12): (1) from the bulk electrolyte to the electrode, (2) within the porous structure of the electrode, (3) from the electrolyte in electrode to the active material, and (4) within the active material itself [100]. The beneficial effects of g‐C_3_N_4_ inclusion are primarily observed in the third step. They also partially improve the second step, thereby conferring an advantage during the discharge stage. Despite the advantages introduced by g‐C_3_N_4_ incorporation, achieving sustained and uniform electrochemical reactions during rapid discharge ultimately depends on the electrode's pore structure. In particular, the second step of Li^+^ transport is strongly governed by porosity and tortuosity. To ensure that the influx of Li^+^ is consistently smooth and rapid, and that the supply rate matches or exceeds the depletion rate, it is essential to optimize these structural parameters of electrode to minimize transport resistance under high‐rate discharge conditions.

The tortuosity of an electrode structure significantly affects lithium‐ion transport pathways, even among electrodes with similar compositions [101]. Given that g‐C_3_N_4_ has a lower density compared to the cathode active material, incorporating it at equivalent electrode densities inevitably alters electrode porosity. Because PTFE fibrillation critically governs the processability and integrity of dry electrodes, all compositions were kneaded under conditions selected to achieve an optimal fibrillation state within a manufacturable process window. Within the g‐C_3_N_4_ composition range investigated here, the practical kneading window did not change substantially, and SEM observation did not reveal a pronounced difference in PTFE fibril morphology among the fabricated electrodes (Figure S13). These results suggest that the deterioration in electrochemical performance at high g‐C_3_N_4_ contents is not primarily caused by a major loss of fibrillation efficiency. Therefore, to elucidate the pore‐structural origin of the performance deterioration, all electrode samples underwent X‐ray computed tomography (CT) imaging, enabling digital reconstruction of their pore structures using Avizo software (Figure S14). The detailed procedures for pore segmentation and reconstruction are outlined in Section 4 and Supporting Information. These reconstructed pore structures allowed quantitative estimation of electrode tortuosity (Figure 5j,k). To independently validate the tortuosity trend, the ionic resistance (R_ion_) of each electrode was also evaluated using symmetric cell EIS, from which tortuosity was calculated as τ = ($R_{\mathrm{ion}}\times A\times \varepsilon \times \sigma$) / d (Figure S15 and Table S12). The EIS‐derived tortuosity values are higher than the CT‐based values by a factor of approximately 2–3, which is attributed to the limited spatial resolution of X‐ray CT applied to the dry electrode: the low‐density carbon/binder domain cannot be distinguished from the empty pore space and is partially recognized as accessible pore volume (Figure S15d), leading to an underestimation of the true tortuosity. Nonetheless, both methods yield a consistent trend across all g‐C_3_N_4_ contents.

As anticipated, the tortuosity values exhibited an overall increasing trend with higher g‐C_3_N_4_ content, indicating that g‐C_3_N_4_ incorporation ultimately hinders Li^+^ diffusion within electrodes. This tortuosity increase explains the inflection in electrochemical performance, despite g‐C_3_N_4_’s beneficial properties. Notably, tortuosity did not uniformly increase with greater g‐C_3_N_4_ content. Specifically, at 0.25% g‐C_3_N_4_, tortuosity decreased slightly before rising again, reaching its maximum at 1% g‐C_3_N_4_, and subsequently decreased at higher concentrations (1.5% and 2%). This non‐linear trend required further investigation.

Electrode density achieved through calendaring, a precision rolling process that compacts the electrode to a specified thickness, is governed by the intrinsic characteristics of the electrode materials. Excessive calendaring pressure can induce structural cracking or a spring‐back effect, where compressed electrodes revert partially to their original thickness over time. This phenomenon becomes pronounced with increasing use of low‐density materials like g‐C_3_N_4_, potentially affecting both porosity and tortuosity [102]. We monitored electrode thickness over 15 days post‐compression at a target density (∼3.0 g/cc), revealing clear spring‐back effects in all samples (Figure S16). Analysis showed a correlation between increased g‐C_3_N_4_ content and greater pore volume expansion over this period (Table S13). These observations confirmed that internal pore structures are dynamically rearranged post‐calendaring, significantly influencing tortuosity.

From an engineering perspective, the pronounced spring‐back phenomenon observed at higher g‐C_3_N_4_ concentrations presents a critical challenge for large‐scale dry electrode manufacturing. The dynamic rearrangement of the internal pore structure not only increases tortuosity but also compromises the mechanical integrity of the thick film. Consequently, mitigating this effect requires careful optimization of calendering pressure and precise management of aging time to stabilize the electrode architecture before electrolyte filling.

To deepen the analysis, we constructed a pore network model from reconstructed pore structures using Avizo software, quantifying both pore sizes and pore‐to‐pore connectivity (Figure S17). Here, the coordination number represents the number of connections a single pore has with adjacent pores. This connectivity directly influences lithium‐ion pathways, critically determining ionic transport efficiency. Analysis indicated that pore connectivity, quantified as the coordination number, decreased with increasing g‐C_3_N_4_ content. Thus, although electrodes containing 1.5% and 2% g‐C_3_N_4_ exhibited the highest pore volume after 15 days due to substantial spring‐back effects, the decrease in pore connectivity counteracted the potential reduction in tortuosity expected from increased porosity. Consequently, tortuosity reflects a complex interaction between pore volume and connectivity.

To further understand how this initial pore network evolves and influences long‐term stability, post‐mortem X‐ray CT and scanning electron microscopy (SEM) were performed on the 0 wt% and 0.5 wt% g‐C_3_N_4_ electrodes after 600 cycles (Figure S18 and Table S14). The 0 wt% electrode exhibited significant structural degradation: pore volume decreased by 8.3% (from 32.08% to 29.42%), tortuosity increased by 39.1% (from 1.1883 to 1.6535), and pore coordination number decreased by 17.7% (from 5.301 to 4.363). This indicates severe pore clogging and loss of ion transport connectivity, consistent with the thick accumulation of byproducts observed in the SEM image (Figure S18h). In contrast, the 0.5 wt% electrode showed comparatively better retention of its pore structure, with a more moderate increase in tortuosity of 16.3% (to 1.3991) and a relatively smaller decrease in coordination number of 9.7% (to 4.412), alongside a comparatively cleaner surface (Figure S18i). This structural preservation is attributed to the reaction uniformity induced by the g‐C_3_N_4_ additive. By promoting uniform reaction kinetics across the electrode, g‐C_3_N_4_ prevents localized over‐utilization and severe side reactions, thereby minimizing the localized accumulation of cathode‐electrolyte interphase (CEI) and electrolyte decomposition byproducts that cause pore clogging. The relatively intact pore network ensures sustained ion transport, directly supporting the improved long‐term capacity retention observed in the 0.5 wt% electrode.

To further verify the long‐term structural stability of the additive, post‐mortem XPS analysis was conducted on the 0.5 wt% g‐C_3_N_4_ electrode after 600 cycles. As shown in Figures S19a and S19b, the characteristic N 1s peaks corresponding to sp^2^ hybridized nitrogen (398.5 eV) and bridging nitrogen (399.5 eV) of the heptazine framework are clearly preserved at their original binding energies. This result confirms that the g‐C_3_N_4_ framework remains chemically persistent and does not undergo severe structural evolution or dissolution during prolonged cycling, thereby sustaining its functional role in facilitating Li^+^ transport. Building upon the confirmed structural persistence of g‐C_3_N_4_, further analysis of the F 1s and O 1s XPS spectra reveals that the additive also modulates the composition and evolution of the cathode‐electrolyte interphase (CEI). The F 1s spectra show a shift toward a more inorganic‐rich interphase in the 0.5 wt% g‐C_3_N_4_ electrode, as evidenced by an enhanced LiF contribution relative to organic fluorine species (Figures S19c and S19d). In addition, the O 1s spectrum of the 0.5 wt% g‐C_3_N_4_ electrode exhibits reduced organic CEI components and a clearer recovery of the metal‐oxygen (M–O) lattice signal compared with the additive‐free electrode (Figures S19e and S19f), consistent with a thinner and more stable CEI. These results suggest that g‐C_3_N_4_ contributes to interfacial stabilization by mitigating excessive electrolyte decomposition, which aligns well with the cleaner electrode surface and preserved pore connectivity observed in the post‐mortem SEM and X‐ray CT analyses (Figure S18).

Overall, these findings highlight the intricate balance between structural integrity, pore connectivity, and Li^+^ transport efficiency, which are all essential for optimizing the electrochemical performance of dry thick‐film electrodes incorporating g‐C_3_N_4_. To identify the optimal conditions leveraging the benefits of g‐C_3_N_4_ without incurring detrimental structural effects, electrodes containing 0.5 wt% g‐C_3_N_4_ were strategically positioned either at the top or bottom layers in electrodes with increased loading (∼42.2 mg/cm^2^, ∼3.59 g/cc) (Figure S20 and Table S15). Performance evaluation at different discharge rates indicated no substantial differences at 0.1C; however, under higher rate conditions (1.0C), electrodes containing g‐C_3_N_4_ clearly exhibited improved discharge capacities. Remarkably, the electrode with g‐C_3_N_4_ positioned at the bottom demonstrated the highest discharge capacity (89.6 mAh/g), surpassing those without g‐C_3_N_4_ (56.9 mAh/g) and g‐C_3_N_4_ positioned at the top (68.5 mAh/g). The power density, based on the nominal voltage, was found to be 1.32 times higher for the electrode with g‐C_3_N_4_ positioned at the bottom compared to that with g‐C_3_N_4_ positioned at the top, and approximately 1.59 times higher than that of the electrode without g‐C_3_N_4_. This outcome underscores the effectiveness of placing g‐C_3_N_4_ at the bottom to facilitate efficient Li^+^ supply deeper into the electrode structure during rapid discharge. In thick electrodes under high‐rate conditions, the top layer near the separator has ready access to the bulk electrolyte, allowing rapid Li^+^ replenishment. In contrast, the bottom layer near the current collector initially relies on Li^+^ present in the locally impregnated electrolyte; once these are consumed, further supply depends on Li^+^ transport through the electrolyte impregnated within the electrode pore network. The inherently high tortuosity of this pathway limits the Li^+^ replenishment rate, which cannot keep pace with the rate of consumption during fast discharge, leading to Li^+^ depletion near the current collector. Placing g‐C_3_N_4_ at the bottom layer directly addresses this transport limitation by accelerating local Li^+^ transport kinetics, thereby mitigating Li^+^ depletion and sustaining a more uniform Li^+^ distribution across the electrode thickness. Conversely, placing g‐C_3_N_4_ at the top layer does not resolve this fundamental supply imbalance; the associated increase in tortuosity further impedes Li^+^ transport toward the bottom, exacerbating the depletion effect. These results confirm the necessity of meticulously optimizing both the content and positioning of g‐C_3_N_4_ to maximize electrochemical performance in thick‐film electrodes.

While the functionally graded dual‐layer architecture demonstrates significant electrochemical improvements at the laboratory scale, its scalability to industrial manufacturing warrants further consideration. From a processing standpoint, the dry electrode fabrication method is inherently compatible with continuous roll‐to‐roll manufacturing. The dual‐layer design can be practically implemented by sequentially laminating two distinct free‐standing dry films before final calendering. This approach holds substantial promise for scaling up to even higher areal loadings (>50 mg/cm^2^), where the strategic placement of g‐C_3_N_4_ at the bottom layer can effectively mitigate the severe Li^+^ depletion that typically plagues ultra‐thick electrodes. However, transitioning this architecture to large‐format pouch or cylindrical cells will require precise engineering controls to ensure uniform interfacial adhesion between the layers and to manage the differential spring‐back behaviors of layers with varying compositions.

### 2.5 Mechanistic Overview and Quantitative Trade‐Off Analysis

The electrochemical performance of the g‐C_3_N_4_ integrated dry thick‐film cathodes is governed by a complex interplay between kinetic enhancements and structural/electronic penalties. To comprehensively understand this mechanism, we propose a dual‐pathway model that dictates the Li^+^ transport and overall capacity at high discharge rates.

First, the incorporation of g‐C_3_N_4_ introduces two primary kinetic benefits: (1) enhanced electrolyte wettability due to its inherent nanoporous structure, which increases the absolute amount of Li^+^ available within the thick electrode, and (2) the formation of transient Li‐N coordination bonds. These Lewis acid‐base interactions effectively lower the Li^+^ desolvation energy barrier, thereby accelerating the charge‐transfer kinetics at the electrode–electrolyte interface. However, these kinetic advantages are counterbalanced by the intrinsic physical properties of g‐C_3_N_4_. As a polymeric semiconductor with a wide bandgap (∼2.7 eV), g‐C_3_N_4_ exhibits poor electrical conductivity [103]. Consequently, increasing its concentration inevitably raises the overall electronic resistance of the electrode. Furthermore, the low density of g‐C_3_N_4_ exacerbates the spring‐back effect during the dry electrode calendering process, leading to dynamic rearrangements of the internal pore structure. While this increases total porosity, it simultaneously reduces pore connectivity, resulting in higher tortuosity that impedes long‐range Li^+^ diffusion [104].

To examine this trade‐off in a compact quantitative form, we fitted the 3C discharge capacity as a function of g‐C_3_N_4_ content with a second‐order polynomial (Figure S21). Polynomial regression and marginal‐effect analyses have been widely used in battery engineering to characterize non‐linear electrochemical behaviors and to identify regime transitions in additive optimization [105, 106]. We note that this fit is not intended to predict the precise location of the experimental capacity maximum. The moderate *R* ^2^ value reflects the asymmetric shape of the experimental capacity‐vs‐content profile, characterized by a steep rise from 0 to 0.5 wt% followed by a gradual decline, which a symmetric quadratic cannot fully reproduce. The fit is therefore used here as a compact empirical description that distinguishes the regime in which the Li–N coordination benefits governs the marginal capacity gain from the regime in which the electronic and pore‐connectivity penalties dominate. As shown in Figure S21a, the capacity is described by C = −58.2x^2^ + 146.4x + 67.6 (*R* ^2^ = 0.804), where C is the 3C discharge capacity (mAh g^−1^) and x is the g‐C_3_N_4_ content in wt%.

The marginal‐effect analysis (dC/dx = −116.4x + 146.4, Figure S21c) identifies a crossover point at 1.26 wt%, beyond which the net derivative of capacity with respect to additive content becomes negative. This crossover marks the transition from the kinetics‐dominated regime, in which the Li–N coordination benefit governs the marginal change in capacity, to the resistance‐dominated regime, in which the electronic and structural penalties dominate. The experimentally observed optimum at 0.5 wt% lies within the kinetics‐dominated regime defined by this crossover, exhibiting a 165.9% capacity increase relative to the baseline (from 58.8 to 156.2 mAh g^−1^) for an electronic resistance increase of only 11.2 ohm (Figure S21d). The specific location of this optimum is determined by the cooperative microstructural and interfacial mechanisms, namely Li–N coordination density, spring‐back, and pore connectivity, as detailed in the preceding sections, rather than by the regression itself. In the higher‐loading regime, the electronic and structural penalties dominate the marginal behavior of the capacity. Consistent with this, a negative linear correlation is observed between electronic resistance and 3C capacity for samples above 0.5 wt% (*R* ^2^ = 0.867, Figure S21b), with a slope of −0.47 mAh g^−1^ per ohm. Taken together, the regression positions the experimental optimum at 0.5 wt% within the kinetics‐dominated regime and frames the deterioration at higher loadings as the consequence of crossing into the resistance‐dominated regime.

To comprehensively understand how these structural rearrangements and the intrinsic properties of g‐C_3_N_4_ collectively govern the electrochemical kinetics, we further analyzed the charge transfer resistance (R_ct_) obtained from EIS measurements (Figure S22a). The R_ct_ values exhibit a distinct non‐linear evolution with increasing g‐C_3_N_4_ content, which can be elucidated by a three‐stage dominant mechanism reflecting the competition between ionic enhancement and electronic blocking (Figure S22b).

In the first stage (≤0.5 wt%), the formation of transient Li‐N bonds effectively lowers the Li^+^ desolvation energy barrier, and this kinetic benefit dominates the system, leading to a decrease in R_ct_ (from 8.22 ohm to 7.47 ohm at 0.25 wt%). In the second stage (0.5 to 1.0 wt%), the intrinsic insulating nature of g‐C_3_N_4_ becomes the rate‐limiting factor. The accumulation of g‐C_3_N_4_ substantially increases the overall electronic resistance of the electrode, hindering the electron transfer step necessary for the interfacial reaction and causing R_ct_ to rise to 8.91 ohm at 1.0 wt%.

Remarkably, in the third stage (1.5 to 2.0 wt%), R_ct_ decreases again, reaching 8.23 ohm and 7.37 ohm, respectively. This re‐decrease is directly driven by the aforementioned spring‐back effect. The expansion in pore volume at high g‐C_3_N_4_ concentrations facilitates electrolyte infiltration, exposing a larger surface area of g‐C_3_N_4_ to the electrolyte and reactivating Li‐N bond formation. This lowers the local interfacial charge transfer resistance. However, this creates a critical paradox: while the local interfacial reaction is accelerated (low R_ct_), the actual high‐rate performance (e.g., 3C discharge capacity) of the 1.5 and 2.0 wt% electrodes falls short of the expectations set by their fast local kinetics, remaining inferior to the 0.5 and 1.0 wt% electrodes (Figure S22d). This discrepancy occurs because the expanded pores suffer from reduced connectivity (coordination number decreases from 5.301 at 0.0 wt% to 4.778 at 1.5 wt% and 4.825 at 2.0 wt%, Figure S22c), and the electronic resistance remains at its peak. Consequently, despite the rapid local kinetics, the long‐range transport of both Li^+^ and electrons is fundamentally restricted, preventing the electrodes from achieving their full potential at high rates. This mechanistic insight confirms that optimizing both the content and positioning of g‐C_3_N_4_ is imperative.

## 3 Conclusions

We successfully fabricated high‐performance dry thick‐film cathodes incorporating g‐C_3_N_4_ as a functional additive. Our findings demonstrate that increasing electrode thickness typically impedes Li^+^ transport, particularly to the lower electrode regions. However, integrating g‐C_3_N_4_ significantly mitigates this issue by enhancing electrolyte wettability and facilitating the formation of Li–N bonds, thereby increasing Li^+^ availability within the electrodes. These improvements notably extended high‐rate discharge capabilities and cycle life, delaying Li^+^ depletion during rapid initial discharge phases. Moreover, Li–N bonding within g‐C_3_N_4_ effectively reduced Li^+^ desolvation energy, thereby decreasing diffusion energy barriers and enhancing discharge kinetics. Despite these significant electrochemical advantages, g‐C_3_N_4_ incorporation inevitably increased electrical resistance and structural tortuosity within electrodes. X‐ray CT and pore network modeling analyses revealed complex, nonlinear relationships between g‐C_3_N_4_ content, pore connectivity, and electrode porosity, strongly influencing Li^+^ transport pathways. Specifically, although higher g‐C_3_N_4_ contents resulted in substantial spring‐back effects and increased porosity, reduced pore connectivity effectively negated the potential improvements in tortuosity arising from enhanced porosity. Consequently, optimal electrochemical performance depends critically on carefully balancing g‐C_3_N_4_ content to ensure efficient Li^+^ transport throughout the electrode structure. Remarkably, despite additive incorporation generally reducing battery energy density, electrodes with optimized g‐C_3_N_4_ content achieved 2.85 times higher power density compared to electrodes without the additive. Thus, this study underscores the importance of fine‐tuning additive functionality and concentration to enhance thick‐film electrode performance and validates, for the first time, the substantial potential of g‐C_3_N_4_ as a novel additive for advanced dry thick‐film cathodes.

## 4 Exprimental Section

### 4.1 Synthesis of Graphitic Carbon Nitride (g‐C_3_N_4_)

The following method was used to synthesize graphitic carbon nitride. 1 g of melamine was stirred in dimethyl sulfoxide to prepare a solution. And, cyanuric acid was stirred in dimethyl sulfoxide. Next, mix the combination of melamine and glucose with cyanuric acid, let it stand for 2 h, perform centrifugation, and wash it three times with deionized water and ethanol. The precipitate was collected, dried at 80°C for 24 h, ground using mortar, and thermal‐treated in a tube furnace at 550°C in a N_2_ atmosphere. To prepare the high‐temperature‐treated g‐C_3_N_4_ control sample (HT‐g‐C_3_N_4_), the as‐synthesized g‐C_3_N_4_ powder was further annealed at 600°C for 1 h under N_2_ atmosphere. The secondary annealing was performed to induce compact densification of the carbon nitride framework and reduce accessible porosity while preserving nitrogen‐containing Li^+^‐coordination sites.

### 4.2 Preparation of the Electrode by Dry Electrode Manufacturing Process

Commercial LiNi_0.8_Co_0.1_Mn_0.1_O_2_ (NCM811 powder, P&O Chemical, Korea) was used as the cathode active material, and carbon black (Super P, Alpha Aesar) was used as the conductive material. Polytetrafluoroethylene (PTFE powder) served as the binder obtained from SANG‐A‐FRONTEC. The dry process for the solvent‐free thick‐film electrode is as follows. The ratio of the electrode material is 96% cathode active material, 2.0% carbon black, and 2.0% binder (PTFE). The materials were placed in a blender, mixed for 3 min, and subsequently underwent a second dispersing and fibrosis process using an agate mortar and pestle for research paper purposes. Based on this process, graphitic carbon nitride (g‐C_3_N_4_) was added as an additive at ratios of 0.0%, 0.25%, 0.5%, 1.0%, 1.5% and 2.0% to the cathode active material, constituting 96% of the mixture. PTFE exhibits fibrous changes when shear stress is applied. The initial fibrous dry electrode was changed from a high to a low gap by passing through a rolling mill machine to achieve tertiary dispersion and secondary fibrosis. Subsequently, a roll press machine was used to control the current electrode density, and the resulting electrode was then attached to the current collector. As a current collector, carbon‐coated aluminum (15 µm) was used to improve adhesive force and conductivity with dry‐electrodes. Electrodes with a current density of 4mAh cm^−2^ were then manufactured. All processes were carried out in a controlled environment with a constant temperature and humidity.

### 4.3 Li Treatment

The fabricated electrode is immersed in the electrolyte (1.15 M LiPF6 in EC: EMC = 3:7 + FEC 10%) for 4 h, subsequently rinsed approximately three times with dimethyl carbonate (DMC) solution, and then dried at room temperature for about 5 h.

### 4.4 Material Characterization (SEM, FTIR, XPS, Pycnometer)

The cross‐sections of the electrodes were observed using high‐resolution field‐emission scanning electron microscopy (UHR FE‐SEM; MCP10). Ion milling was conducted using a cooling cross‐section polisher (MCP10) to obtain cross‐sectional images. Energy‐dispersive X‐ray spectroscopy (EDS) was used to map nitrogen in the electrodes. To confirm the result that nitrogen attracts lithium ions to form bonds, g‐C_3_N_4_ sheets were fabricated, and Attenuated Total Reflection Fourier Transform Infrared (ATR FT‐IR) spectroscopy analysis was adopted. Polyvinylidene fluoride (PVDF 6020), composed of NMP 5 g and g‐C_3_N_4_ 0.1 g, was mixed using a planetary mixer (AR‐100; Thinky Co.), coated on an aluminum current collector, and vacuum dried at 120°C overnight to prepare sheets. Samples treated with electrolyte were compared with untreated samples. To confirm the result that g‐C_3_N_4_ added to the electrode attracts lithium ions to form bonds, the XPS (Thermo Fisher Scientific K‐Alpha^+^, NFEC‐2020‐09‐265067) analysis method was used. After preparing electrodes with different ratios, samples treated with electrolyte were analyzed and compared with untreated samples. The true densities of g‐C_3_N_4_ and the high‐Ni cathode material were measured using a gas pycnometer (Ultrapyc 5000, Anton Paar). All samples were dried and degassed prior to measurement, and the analysis was performed at room temperature under stable thermal conditions.

### 4.5 Assembled of Half Coin Cell

The cells manufactured for the electrochemical performance evaluation were assembled in a 2032 coin‐type format. A polyethylene separator (SB16C, W scope), with a thickness of 14 µm was used, and 1.15 M LiPF6 in EC(Ethylene Carbonate): EMC(Ethyl Methyl Carbonate) = 3:7 + FEC(Fluoroethylene Carbonate) 10% electrolyte. A half cell was assembled using lithium metal (300 µm, Honjo Metal Co., Japan) as the anode.

### 4.6 Electrochemical Measurements

The performance of the electrode using the g‐C_3_N_4_ additive was evaluated using a multichannel battery tester (WBCS Battery Cycler, Wonatech Co.) in a room temperature chamber. During the formation test, charging and discharging were conducted at a current rate of 0.1C and measured at a cutoff voltage of 2.5 V–4.3 V. The rate performance was assessed by discharging the battery at various rates (0.1C, 0.2C, 0.5C, 1C, 2C, and 3C), while maintaining a fixed charge rate of 0.1C. Cycle performance was evaluated by charging and discharging at a rate of 1C for 100 cycles. Both rate performance and cycle performance were measured within the cut‐off voltage range of 2.75 V–4.3 V.

For the full‐cell evaluation, single‐layer pouch cells were assembled using the prepared thick‐film cathodes and artificial graphite anodes. The cathode electrodes were cut to a size of 3.0 cm x 4.2 cm (12.6 cm^2^) with an areal capacity of 4.0 mAh/cm^2^. The anode was fabricated using artificial graphite (ShanShan) with a specific capacity of 345 mAh/g (at 0.2C) and a composition of 94:1:5 (active material: Super P:CMC/SBR, wt%). The anode was designed with an areal loading of approximately 12.99 mg/cm^2^ to achieve an N/P ratio of 1.12 (based on the active electrode area of 3.0 × 4.2 cm), and the anode composite layer was calendered to a density of 1.5 g/cc. The anode electrode dimensions were 3.1 × 4.3 cm to provide a 1 mm overhang on each side relative to the cathode. The pouch cells were assembled in a dry room, and a polyethylene separator (SB16C, W‐scope, 14 um) was placed between the cathode and anode. The electrolyte used was 1.15 M LiPF_6_ in EC:EMC (3:7 v/v) with 10 wt% FEC. To ensure proper wetting of the thick electrodes while avoiding excessive electrolyte flooding, the electrolyte amount was strictly controlled at an electrolyte‐to‐capacity (E/C) ratio of 3.0 g/Ah (approximately 150 mg of electrolyte per cell). After electrolyte injection and vacuum sealing, the pouch cells were allowed to rest for 24 h to ensure complete electrolyte permeation before electrochemical testing. The detailed design specifications of the pouch full‐cells are summarized in Table S10.

### 4.7 High Resolution X‐Ray Nanotomography

Electrode structure analysis was carried out using a high‐resolution 3D X‐ray nanotomography (XRM, ZEISS Xradia 810 Ultra) to visualize and evaluate the internal 3D microstructure. A total of six electrodes with varying g‐C_3_N_4_ composition ratios were fabricated, each exhibiting a current density of 4 mAh cm^−2^ and a composite layer density of 3.0 g cm^−3^. These samples were subjected to X‐ray scanning with continuous rotation at defined angular intervals. Specifically, the X‐ray nanotomography was performed at an optical magnification of 40×, with an acceleration voltage of 100.22 kV and a beam current of 90 µA. The exposure time was set to 8 s per projection. The resulting volumetric dataset exhibited an isotropic voxel size of 0.3192 µm. This scanning process enabled the collection of a series of 2D radiographic images from multiple perspectives, which were reconstructed into 3D microstructural images through tomographic reconstruction. The resulting 3D images were processed and visualized using commercial software (Avizo, Thermo Fisher Scientific). From these reconstructions, porosity and tortuosity were extracted through quantitative image analysis.

### 4.8 Symmetric Cell Fabrication

To evaluate the ionic resistance (R_ion_) and tortuosity (τ) of the electrodes, symmetric cells were assembled by placing two identical electrodes (3.0 cm x 4.2 cm) face‐to‐face with a polypropylene separator (Celgard 2400) between them. The cells were filled with 1.15 M LiPF_6_ in EC:EMC (3:7 v/v) with 10 wt% FEC.

### 4.9 Preparation of the Dual Layer Electrode by Dry Electrode Manufacturing Process

Dual‐layer electrodes were fabricated by stacking two independently prepared dry electrodes, each manufactured following the previously described dry electrode process. Two types of single‐layer electrodes were prepared: one with and one without 0.5 wt% g‐C_3_N_4_ added to the cathode active material. After preparing the individual electrodes, two sheets were carefully laminated by overlaying them in the desired configuration (0.0%/0.0%, 0.5%/0.0%, or 0.0%/0.5% g‐C_3_N_4_). The laminated sheets were subsequently passed through a roll press to promote mechanical bonding between the layers and to achieve a final electrode thickness of approximately 113 µm. Throughout the process, particular attention was given to maintaining the target electrode density (∼3.6 g/cc) and ensuring uniform adhesion between the layers. Carbon‐coated aluminum foil (15 µm) was used as the current collector to enhance electrical conductivity and interfacial adhesion with the dry electrode.

## AI Statement

During the preparation of this manuscript, the authors used ChatGPT, developed by OpenAI, solely for English‐language editing, including grammar correction and improvements in clarity and readability. The use of ChatGPT was limited to language editing of the English text throughout the manuscript. ChatGPT was not used to generate scientific content; formulate hypotheses; analyze or interpret experimental data; prepare or modify figures, tables, or supporting information; generate code; or draw scientific conclusions. All AI‐assisted language revisions were critically reviewed and approved by the authors, who take full responsibility for the accuracy and final content of the manuscript.

## Conflicts of Interest

The authors declare no conflicts of interest.

## Supporting information




**Supporting File**: exp270207‐sup‐0001‐SuppMat.docx.

## Data Availability

The data that support the findings of this study are available from the corresponding author upon reasonable request.
