# Supplementary material for: Architecture‐Interface Co‐Design of g‐C3N4 Driven Dry Thick Cathodes Enabling Fast Li‐Ion Transport
Source: Exploration (Beijing). 2026 Aug 11;6(4):70207. doi: 10.1002/exp2.70207 (PMC13461870; doi:10.1002/exp2.70207)
Supplement: Supplementary file 1 [file EXP2-6-70207-s001.docx]

Supporting Information

Architecture–Interface Co-Design of g-C_3_N_4_ Driven Dry Thick Cathodes Enabling Fast Li-Ion Transport

**Figure S1.** Schematic illustration and photographs comparing electrode microstructures according to wet (PVDF binder) and dry (PTFE binder) fabrication processes and photographs for fabarication process of the electrode via dry fabrication processes. a) Comparative schematic highlighting binder morphology and material distribution differences between the two processes. b) Mixed electrode powder prior to fibrillation. c) Electrode mixture transformed into a malleable dough through PTFE fibrillation. d) Thin electrode sheet formation via roll pressing. e) Completed dry electrode laminated onto an aluminum foil current collector.

**Table S1.** Information on electrodes with g-C_3_N_4_ added at various ratios used in electrochemical performance analysis

| **Sample** | **Loading level**  **(mg/cm^2^)** | **Thickness**  **(um)** | **Electrode density**  **(g/cc)** |
| --- | --- | --- | --- |
| g-C_3_N_4_ 0.0% | 21.30 | 68 | 3.01 |
| g-C_3_N_4_ 0.25% | 21.69 | 69 | 3.02 |
| g-C_3_N_4_ 0.5% | 21.50 | 68 | 3.04 |
| g-C_3_N_4_ 1.0% | 21.31 | 69 | 2.96 |
| g-C_3_N_4_ 1.5% | 21.56 | 69 | 3.00 |
| g-C_3_N_4_ 2.0% | 21.45 | 68 | 3.03 |

**Wetting angle measurement**

The contact angle analysis was conducted to determine whether g-C_3_N_4_ enhances the wettability of the electrode. Dry-electrodes with varying proportions of g-C_3_N_4_ (0.0%, 0.25%, 0.5%, 1.0%, 1.5%, and 2.0%) were prepared, and the same electrolyte used for half-cell assembly was used as the solvent for contact angle measurements. The measurements were performed at room temperature, and drop images were recorded using a video camera attached to a microscope equipped with the contact angle equipment.

**Figure S2.** a) Comparison of the wettability between electrodes with and without g-C_3_N_4_ additives, and the electrolyte (1.15 M LiPF_6_ in EC:EMC=3:7 + FEC 10%). b) The contact angle between electrolyte and electrodes with and without g-C_3_N_4_.

**Figure S3.** A photograph of a dry-processed sheet composed of PTFE (10%) and g-C₃N₄ (90%), exhibiting a uniform and smooth surface, demonstrating successful fabrication of a flexible, free-standing film.

**Figure S4.** Li1s XPS spectrum before and after electrolyte impregnation treatment of electrodes containing or without g-C_3_N_4_ (0.0%, 0.25%, 0.5%, 1.0%, 1.5% and 2.0%).

**Table S2.** Possible compositions and their binding energies in the Li 1s and N 1s spectra.

| **Peak** | **Spectral line** | **Assignment** |
| --- | --- | --- |
| Li 1s | 55.0 | LiOH/Li_2_CO_3_ |
|  | 55.2 | Li-N |
|  | 55.8-56.0 | LiF |
| N 1s | 398.5 | Sp^2^ N (triazine rings) |
|  | 398.7 | Li-N |
|  | 399.5 | Bridging N (N-(C)_3_) |
|  | 400.8 | N-H |

**Table S3.** Relative peak area ratios (%) of the N 1s XPS spectra before and after Li treatment.

| Sample | 0.25% | | | 0.5% | | | 1.0% | | | 1.5% | | | 2.0% | | |
| --- | --- | --- | --- | --- | --- | --- | --- | --- | --- | --- | --- | --- | --- | --- | --- |
| Li treatment | Before | | After | Before | | After | Before | | After | Before | | After | Before | | After |
| Sp^2^ N / 398.5 eV (%) | 38.7 | 18.0 | | 43.1 | 16.2 | | 39.2 | 12.3 | | 46.0 | 11.4 | | 38.8 | 11.5 | |
| Li-N / 398.7 eV (%) | 0 | 9.6 | | 0 | 11.9 | | 0 | 8.6 | | 0 | 8.2 | | 0 | 10.0 | |
| Bridging N / 399.5 eV (%) | 34.6 | 28.9 | | 25.3 | 36.9 | | 28.3 | 39.5 | | 30.7 | 40.9 | | 29.2 | 37.2 | |
| N-H / 400.8 eV (%) | 26.7 | 43.5 | | 31.7 | 34.9 | | 32.5 | 39.6 | | 23.3 | 39.5 | | 32.0 | 41.3 | |

**Table S4.** Relative peak area ratios (%) of the Li 1s XPS spectra after Li treatment.

| Sample | 0.0% | 0.25% | 0.5% | 1.0% | 1.5% | 2.0% |
| --- | --- | --- | --- | --- | --- | --- |
| LiOH/Li_2_CO_3_ /55.0 eV (%) | 38.6 | 37.3 | 34.4 | 29.6 | 24.1 | 17.0 |
| Li-N / 55.2 eV (%) | 0 | 26.0 | 30.3 | 26.7 | 31.2 | 30.6 |
| LiF / 55.8-56.0 eV (%) | 61.4 | 36.7 | 35.3 | 43.7 | 44.7 | 52.4 |

**Impedance measurement method**

Electrochemical impedance spectroscopy (EIS) measurements were conducted using a potentiostat (VMP3, Bio-Logic) over a frequency range from 1 MHz to 10 mHz with an AC voltage amplitude of 5 mV. For the pristine half-cells (Figure S5), EIS was measured at various temperatures (283.15 K to 323.15 K) before any electrochemical cycling to determine the activation energy. For the symmetric cells (Figure S15), EIS was measured at 25 °C using non-blocking symmetric coin cells (cathode/separator/cathode) to calculate the effective ionic resistance (R_ion_) and tortuosity of the electrodes. All EIS data were fitted using ZView software.

**Figure S5.** Nyquist plots for half cells after formation using electrodes with a) g-C_3_N_4_ 0.0%, b) 0.25%, c) 0.5%, d) 1.0%, e) 1.5%, and (f) 2.0% ratios, at various temperatures (283.15 K, 293.15 K, 303.15 K, 313.15 K, and 323.15 K) representing resistance.

**Table S5.** EIS fitting results (R_s_ and R_ct_) of the pristine half-cells at various temperatures.

| Sample | 0.0% | | 0.25% | | 0.5% | | 1.0% | | 1.5% | | 2.0% | |
| --- | --- | --- | --- | --- | --- | --- | --- | --- | --- | --- | --- | --- |
| T (K) | R_s_ (Ω) | R_ct_ (Ω) | R_s_ (Ω) | R_ct_ (Ω) | R_s_ (Ω) | R_ct_ (Ω) | R_s_ (Ω) | R_ct_ (Ω) | R_s_ (Ω) | R_ct_ (Ω) | R_s_ (Ω) | R_ct_ (Ω) |
| 283.15 | 4.81 | 12.35 | 2.69 | 8.76 | 2.46 | 6.76 | 4.69 | 10.42 | 3.16 | 8.89 | 3.68 | 8.61 |
| 293.15 | 4.71 | 7.80 | 2.42 | 7.99 | 1.98 | 7.07 | 4.13 | 9.34 | 2.71 | 8.28 | 3.24 | 8.80 |
| 303.15 | 4.27 | 8.22 | 2.09 | 7.47 | 1.84 | 7.91 | 3.88 | 8.91 | 2.49 | 8.23 | 3.09 | 7.37 |
| 313.15 | 4.03 | 3.45 | 2.14 | 2.45 | 1.82 | 4.65 | 3.74 | 2.85 | 2.37 | 3.86 | 2.25 | 2.68 |
| 323.15 | 3.29 | 0.79 | 2.24 | 0.87 | 1.52 | 2.23 | 3.45 | 3.31 | 2.34 | 2.29 | 2.48 | 1.13 |

**Table S6**. Comparison of activation energy values according to the relationship between R_ct_ values at various temperatures for electrodes with or without g-C₃N₄ additives.

|  | **g-C_3_N_4_ 0.0%** | **g-C_3_N_4_ 0.25%** | **g-C_3_N_4_ 0.5%** | **g-C_3_N_4_ 1.0%** | **g-C_3_N_4_ 0.5%** | **g-C_3_N_4_ 2.0%** |
| --- | --- | --- | --- | --- | --- | --- |
| 1000/T (1/K) | ln(T/R_ct_) | | | | | |
| 3.5316 | 3.1324 | 3.4755 | 3.7352 | 3.3022 | 3.4612 | 3.4934 |
| 3.4112 | 3.6265 | 3.6027 | 3.7253 | 3.4460 | 3.5669 | 3.5060 |
| 3.2986 | 3.6077 | 3.7033 | 3.6461 | 3.5271 | 3.6064 | 3.7168 |
| 3.1933 | 4.5092 | 4.8520 | 4.2093 | 4.6989 | 4.3957 | 4.7591 |
| 3.0945 | 6.0157 | 5.9219 | 4.9780 | 4.5810 | 4.9500 | 5.6547 |
| Activation energy  (kJ mol^-1^) | 49.8117 | 46.0529 | 22.0537 | 24.0300 | 28.5162 | 41.7704 |

**Electrical conductivity measurements of the electrodes**

The electrode resistance measurement system (RM2610, HIOKI Corp., Japan) was used to measure the interface resistance between the carbon-coated aluminum current collector and the composite layer fabricated via a dry process, as well as the volume resistivity within the electrodes. The electrical resistance is measured at six points per single electrode sheet. The parameters that need to be inputted into this device are the current collector thickness, composite layer thickness, and resistivity of the current collector. The parameters that need to be inputted into this device are the current collector thickness, composite layer thickness, and resistivity of the current collector. After that, a constant current of the electrode sheet flows, and the potential distribution generated on the surface is induced between several pins. Several different pins are located on the electrode surface between the current pins to model the electrode sheet so that the device calculates the potential generated on the surface. The calculation potential is repeated until the surface distribution calculated using composite layer resistance and interfacial resistance as variables matches the measured value, and the result is output.

**Figure S6.** The electrical conductivity of the electrodes with different g-C_3_N_4_ contents.

**Figure S7.** Cyclic voltammetry (CV) curves at various scan rates ranging from 0.05 mV s^-1^ to 2.0 mV s^-1^ across different voltage ranges. CVs were recorded for electrodes with g-C_3_N_4_ additives at concentrations of a) 0.0%, b) 0.25%, c) 0.5%, d) 1.0%, e) 1.5%, and f) 2.0%, over a voltage range of 2.75 to 4.3 V at room temperature, revealing distinct observations of oxidation-reduction peaks.

**Table S7.** Comparison of capacitance effect values according to the relationship between scan rate and oxidation-reduction peak currents in half cells with electrodes containing or lacking g-C₃N₄.

|  | **g-C_3_N_4_ 0.0%** | **g-C_3_N_4_ 0.25%** | **g-C_3_N_4_ 0.5%** | **g-C_3_N_4_ 1.0%** | **g-C_3_N_4_ 0.5%** | **g-C_3_N_4_ 2.0%** |
| --- | --- | --- | --- | --- | --- | --- |
| log(ν (mV s^-1^)) | log(i (mA)) | | | | | |
| -1.301 | 0.3433 | 0.3216 | 0.3313 | 0.3985 | 0.3077 | 0.3530 |
| -1.000 | 0.5649 | 0.5545 | 0.5616 | 0.6416 | 0.5383 | 0.5866 |
| -0.699 | 0.7967 | 0.7883 | 0.7959 | 0.8910 | 0.7749 | 0.8295 |
| -0.301 | 1.0517 | 1.0483 | 1.0494 | 1.1626 | 1.0437 | 1.1101 |
| 0.000 | 1.1392 | 1.1479 | 1.1571 | 1.2557 | 1.1574 | 1.2352 |
| 0.301 | 1.1502 | 1.1797 | 1.1984 | 1.2656 | 1.1915 | 1.2684 |
| b value | 0.5287 | 0.5563 | 0.5601 | 0.5668 | 0.5752 | 0.5978 |

**Figure S8.** The corresponding charge/discharge profile at different rates (0.1C, 0.2C, 0.5C, 1.0C, 2.0C, and 3.0C) of half cells assembled with g-C_3_N_4_ electrodes at a) 0.0%, b) 0.25%, c) 0.5%, d) 1.0%, e) 1.5%, and f) 2.0%.

**Table S8.** Comparison of electrochemical performance at 3C of half cells using Li metal as an anode to confirm the actual performance of electrodes to which g-C_3_N_4_ is added at a ratio of 0.0%, 0.25%, 0.5%, 1.0%, 1.5% and 2.0%.

| **Sample** | **3C_Specific capacity**  **(mAh/g)** | **Norminal voltage**  **(V)** | **Electrode**  **power density**  **(W/kg)** |
| --- | --- | --- | --- |
| g-C_3_N_4_ 0.0% | 59.3 | 3.25 | 578 |
| g-C_3_N_4_ 0.25% | 93.6 | 3.40 | 955 |
| g-C_3_N_4_ 0.5% | 156.7 | 3.51 | 1,651 |
| g-C_3_N_4_ 1.0% | 148.3 | 3.49 | 1,552 |
| g-C_3_N_4_ 1.5% | 141.0 | 3.46 | 1,463 |
| g-C_3_N_4_ 2.0% | 137.1 | 3.47 | 1,427 |

**Control experiment using high-temperature-treated g-C_3_N_4_ to distinguish wettability and Li–N coordination effects**

To distinguish the contribution of Li–N-coordination-assisted interfacial kinetics from simple electrolyte-wettability enhancement, high-temperature-treated g-C**_3_**N**_4_** (HT- g-C**_3_**N**_4_**) was prepared by secondary annealing of the as-synthesized g-C**_3_**N**_4_** at 600 °C for 1 h under N**_2_**. As shown in Figure S9a, HT- g-C**_3_**N**_4_** exhibited a compact and densified morphology with reduced open porosity compared with pristine g-C**_3_**N**_4_**. N**_2_** adsorption/desorption analysis further confirmed the substantial suppression of accessible porosity after high-temperature treatment (Figure S9b and c). The BET surface area decreased from 98.8 to 25.7 m² g⁻¹, and the total pore volume decreased from 1.06 to 0.25 cm³ g⁻¹. The BJH main pore diameter also decreased from 101.02 to 67.2 nm. Consistently, the 0.5 wt% HT- g-C**_3_**N**_4_** electrode showed a contact angle of 42.67°, which was close to that of the additive-free electrode (43.98°) and higher than that of the pristine 0.5 wt% g-C**_3_**N**_4_** electrode (40.36°), confirming that the electrolyte-wettability contribution was strongly weakened by high-temperature treatment (Figure S9e).

Despite this weakened wettability, the N 1s XPS spectra after Li treatment still showed the Li–N component in the HT- g-C**_3_**N**_4_**-containing electrode, indicating that nitrogen-based Li^+^ coordination was retained after high-temperature treatment (Figure S9d). However, the decrease in accessible surface area is expected to reduce the number of exposed Li–N coordination sites available for interaction with Li^+^. To quantify this effect, the accessible surface per electrode mass was estimated by multiplying the nominal additive loading by the BET surface area of each additive (Table S9). This value is used as a simple comparative descriptor of the electrolyte-accessible g-C**_3_**N**_4_** interface, rather than an absolute quantification of Li–N coordination sites. Although the nominal amount of HT- g-C**_3_**N**_4_** in the 0.5 wt% HT- g-C**_3_**N**_4_** electrode was twice that of pristine g-C**_3_**N**_4_** in the 0.25 wt% g-C**_3_**N**_4_** electrode, its accessible surface index was only 0.129 m² g⁻¹ electrode, corresponding to approximately 52% of that of the pristine 0.25 wt% g-C**_3_**N**_4_** electrode (0.247 m² g⁻¹ electrode). This explains why the 0.5 wt% HT- g-C**_3_**N**_4_** electrode exhibited lower C-rate performance than the pristine 0.25 wt% g-C_3_N_4_ electrode, despite the higher nominal additive content.

Nevertheless, the 0.5 wt% HT- g-C**_3_**N**_4_** electrode still outperformed the additive-free electrode under high-rate discharge (Figure S9f). These results indicate that the improved rate capability cannot be explained solely by electrolyte wettability. Rather, Li–N coordination provides a kinetic contribution beyond simple wettability enhancement, while the full benefit of pristine g-C**_3_**N**_4_** arises from the cooperative action of electrolyte accessibility and exposed Li–N coordination sites.

**Figure S9.** Control experiment using high-temperature-treated g-C_3_N_4_ (HT-g-C_3_N_4_) to distinguish electrolyte-wettability effects from Li–N-coordination-assisted interfacial kinetics. a) SEM image of HT-g-C_3_N_4_ showing compact and restacked morphology after secondary annealing at 600 °C for 1 h under N_2_. b) N_2_ adsorption/desorption isotherms of pristine g-C3N4 and HT- g-C_3_N_4_. The inset table summarizes the BET surface area, total pore volume, and BJH main pore diameter. c) BJH pore-size distribution curves of pristine g-C_3_N_4_ and HT-g-C_3_N_4_. d) N 1s XPS spectra of the HT-g-C_3_N_4_-containing electrode before and after Li treatment, confirming the retention of Li–N coordination after high-temperature treatment. e) Contact angles of the additive-free electrode, pristine 0.5 wt% g-C_3_N_4_ electrode, and 0.5 wt% HT-g-C_3_N_4_ electrode. f) C-rate performance comparison of the corresponding electrodes.

**Table S9.** Accessible surface index of pristine g-C_3_N_4_ and HT-g-C_3_N_4_ electrodes.

| **Sample** | **Loading**  **(g/g)** | **BET surface area**  **(m^2^/g)** | **Accessible surface per electrode mass**  **(m^2^/g)** |
| --- | --- | --- | --- |
| g-C_3_N_4_ 0.25% | 0.0025 | 98.8 | 0.247 |
| HT-g-C_3_N_4_ 0.5% | 0.0050 | 25.7 | 0.129 |
| g-C_3_N_4_ 0.5% | 0.0050 | 98.8 | 0.494 |

**Table S10.** Pouch full-cell design specifications.

| **Cell Parameter** | | **Value** | |
| --- | --- | --- | --- |
| Cell format | | Single-layer pouch cell | |
| Separator | | PE (SB16C, W-scope), 14um | |
| Electrolyte | | 1.15 M LiPF_6_ in EC:EMC (3:7 v/v) + 10 wt% FEC | |
| E/C ratio | | 3.0 g/Ah | |
| Voltage window | | 2.5 - 4.25 V | |
| N/P ratio | | 1.12 (based on 3.0 x 4.2 cm) | |
| **Parameter** | **Cathode** | | **Anode** |
| Active material | LiNi_0.8_Co_0.1_Mn_0.1_O_2_  (NCM811, P&O Chemical, Korea) | | Artificial graphite (ShanShan) |
| Composition (AM:CB:Binder, wt%) | 96:2:2 | | 94:1:5 |
| Conductive additive | Super P | | Super P |
| Binder | PTFE | | CMC/SBR (2:3) |
| Current collector | Carbon-coated Al foil | | Cu foil |
| Reference specific capacity (0.2C) | 205 mAh/g | | 345 mAh/g |
| Areal capacity | 4.0 mAh/cm^2^ | | 4.48 mAh/cm^2^ |
| Areal loading | 19.51 mg/cm^2^ | | 12.99 mg/cm^2^ |
| Electrode density | 3.0 g/cc | | 1.5 g/cc |
| Electrode dimensions | 3.0 x 4.2 cm | | 3.1 x 4.3 cm |

**Laser induced breakdown spectroscopy measurement method**

To investigate the charge and discharge processes of chemical elements within lithium batteries depending on the contents of g-C_3_N_4_, we utilized Laser Ablation-Induced Breakdown Spectroscopy (LA-LIBS) with the Applied Spectra J200 Tandem system (NFEC-2022-02-276618). LA-LIBS provided the distribution of lithium elements throughout the entire electrode during repeated electrochemical cycles. To perform chemical mapping at each layer, we systematically examined a 1.5 x 1.5 mm² area by conducting laser sampling on a 7 x 7 grid. We used a 266 nm pulse laser for the experiments, and the laser spot size, set at 200 μm in diameter, was determined by the instrument's software. Elemental depth profiling was carried out layer by layer, covering a total of 150 layers. The depth of the laser ablation craters, which formed after each laser pulse, was approximately ~ 0.5 μm, a dimension influenced by the electrode's porosity and composition.

**Figure S10.** Laser-induced breakdown spectroscopy (LIBS) mapping of dry electrodes, confirming the spatial homogeneity of Li^+^ reactivity across both the upper and lower regions of the electrode. Mapping images of electrodes with and without g-C₃N₄ were obtained prior to rate capability testing: a) bare electrode (g-C₃N₄ 0.0%) and b) g-C₃N₄ 2.0%. c) Electrode surface before LIBS analysis and d) electrode surface after LIBS analysis, showing laser-induced damage spots.

**Figure S11.** Photographic comparison of 0.5 g samples of high-Ni cathode material (NCM) and g-C₃N₄.

| **Sample** | **True density (g/cc)** |
| --- | --- |
| g-C_3_N_4_ | 1.68 |
| High-Ni material | 4.53 |

**Table S11.** True densities of g-C₃N₄ and high-Ni cathode material measured using a gas pycnometer.

**Figure S12.** Schematic illustration of the Li⁺ transport pathways during insertion into the electrode: (1) diffusion of Li⁺ from the bulk electrolyte to the electrode surface, (2) diffusion of Li⁺ through the porous structure of the electrode, (3) diffusion of Li⁺ from the electrolyte-filled pores to the surface of the active material (NCM), (4) solid-state diffusion of Li⁺ within the active material particles.

**Figure S13.** Representative SEM images of dry electrodes containing a) 0, b) 0.25, c) 0.5, d) 1.0, e) 1.5, and f) 2.0 wt% g-C_3_N_4_ after kneading and electrode fabrication. PTFE fibrils are observed across all compositions without a pronounced composition-dependent deterioration in fibril morphology.

**Figure S14.** 3D pore structure analysis of thick-film electrodes. a) Representative 3D X-ray microscopy image of the electrode. b) Image processing sequence for pore segmentation from the raw 3D X-ray image, showing the conversion of grayscale data into a binarized and segmented pore network. c-h) Reconstructed 3D pore structures of electrodes containing c) 0.0%, d) 0.25%, e) 0.5%, f) 1.0%, g) 1.5%, and h) 2.0% g-C₃N₄, obtained using Avizo software.

**Tortuosity Calculation from Symmetric Cell EIS**

The ionic resistance (R_ion_) of each electrode was determined from the Nyquist plots of symmetric cells (Figure S15a). In a symmetric cell, two identical electrodes are assembled face-to-face, and the resulting EIS response follows a transmission-line behavior. The high-frequency real-axis intercept (R_H_) represents the total ohmic resistance of the cell, while the low-frequency linear region, when extrapolated to the real axis, yields the intercept R_L_. As illustrated in Figure S15b, the ionic resistance is extracted as:

**R_ion_ = 3** $\times$ **(R_L_-R_H_)**

The factor of 3 is derived from the analytical solution of the transmission-line model for a symmetric cell, in which the low-frequency real-axis intercept equals R_H_ + R_ion_/3.^(1-3)^ The tortuosity ($\tau$) was then calculated using the following equation:

$$\tau=\frac{R_{ion}\times A\times\varepsilon\times\sigma}{d}$$

The following constants and parameters were used in the calculation. The electrode area (A) was 12.6 cm^2^ (3.0 cm x 4.2 cm). The ionic conductivity of the electrolyte ($\sigma$) was 8.7 x 10^-3^ S/cm, measured for 1.15 M LiPF_6_ in EC:EMC (3:7) with 10 wt% FEC at 25 ^o^C. The electrode porosity ($\varepsilon$) and coating thickness (d) are sample-dependent values measured after 15 days; d was determined by subtracting the 15 um Al current collector thickness from the total electrode thickness. The calculated R_ion_ and $\tau$ values for all samples are summarized in Table S11, and the corresponding tortuosity bar chart is shown in Figure S15c.

**Figure S15.** Tortuosity analysis of electrodes with different g-C_3_N_4_ contents. a) Nyquist plots of symmetric cells. The dashed line indicates the real axis. b) Nyquist plots of electrode with 0% g-C_3_N_4_ contents and schematic illustration of the method for extracting R_H_ and R_L_ from the Nyquist plot; R_ion_ = 3(R_L_ - R_H_). c) Tortuosity values calculated from symmetric cell EIS. d) Representative cross-sectional X-ray CT image of the dry electrode, showing NMC active material particles (bright) and the surrounding pore space (dark). The carbon/binder domain is not distinguishable from the pore space due to its low density and sub-resolution feature size.

**Table S12.** Parameters and tortuosity values calculated from symmetric cell EIS.

| Sample | R_H_ (Ω) | R_L_ (Ω) | R_L_ − R_H_ (Ω) | R_ion_ (Ω) | d (μm) | ε (%) | τ |
| --- | --- | --- | --- | --- | --- | --- | --- |
| 0.0% | 0.73681 | 0.981 | 0.24419 | 0.7326 | 72 | 31.92 | 3.5603 |
| 0.25% | 0.57389 | 0.7839 | 0.21001 | 0.6300 | 71 | 32.08 | 3.1204 |
| 0.5% | 0.66637 | 0.9117 | 0.24533 | 0.7360 | 74 | 32.74 | 3.5696 |
| 1.0% | 0.64351 | 0.9 | 0.25649 | 0.7695 | 72 | 32.46 | 3.8029 |
| 1.5% | 0.7317 | 0.9788 | 0.2471 | 0.7413 | 74 | 33.27 | 3.6535 |
| 2.0% | 0.73888 | 0.9934 | 0.25452 | 0.7636 | 72 | 31.92 | 3.711 |

**Figure S16.** a) Changes in electrode density of dry electrodes measured over a period of 15 days. b–g) Measurements of weight and thickness changes of electrodes containing varying g-C₃N₄ contents: b) 0.0%, c) 0.25%, d) 0.5%, e) 1.0%, f) 1.5%, and g) 2.0%, clearly showing thickness recovery (spring-back effect) over time. The initial and subsequent thicknesses were measured using a thickness gauge with a resolution of 1 µm. The thickness of the Al current collector used was 15 µm.

**Table S13.** Electrode loading levels, thicknesses, and pore volumes measured 1 day and 15 days after calendaring.

| **Sample** | **Loading level**  **(mg/cm^2^)** | **Thickness (um)** | | **Pore volume (%)** | | **∆ V (%)** |
| --- | --- | --- | --- | --- | --- | --- |
|  |  | 1 day | 15 day | 1 day | 15 day |  |
| g-C_3_N_4_ 0.0% | 20.06 | 84 | 86 | 30.00 | 32.08 | 6.94 |
| g-C_3_N_4_ 0.25% | 20.05 | 84 | 87 | 29.72 | 32.74 | 10.15 |
| g-C_3_N_4_ 0.5% | 20.78 | 84 | 87 | 29.44 | 32.46 | 10.28 |
| g-C_3_N_4_ 1.0% | 19.89 | 84 | 87 | 28.87 | 31.92 | 10.57 |
| g-C_3_N_4_ 1.5% | 20.22 | 85 | 89 | 29.48 | 33.27 | 12.86 |
| g-C_3_N_4_ 2.0% | 20.15 | 85 | 89 | 28.93 | 32.74 | 13.18 |

**Figure S17.** a-f) Reconstructed 3D pore network models of electrodes containing a) 0.0%, b) 0.25%, c) 0.5%, d) 1.0%, e) 1.5%, and f) 2.0% g-C₃N₄, obtained from X-ray CT images using Avizo software. The pore structures are color-coded according to the equivalent pore radius. g) Pore network coordination numbers extracted from the reconstructed models, representing the average number of direct connections each pore has with neighboring pores. As the g-C₃N₄ content increased, the coordination number gradually decreased, indicating reduced pore connectivity and suggesting more tortuous ion transport pathways.

**Figure S18.** Post-mortem X-ray CT and SEM analysis of electrodes after 600 charge-discharge cycles. a-c) 3D pore structure reconstruction a), tortuosity distribution map b), and pore network visualization c) of the 0 wt% g-C_3_N_4_ electrode after 600 cycles. d-f) Corresponding 3D pore structure reconstruction d), tortuosity distribution map e), and pore network visualization f) of the 0.5 wt% g-C_3_N_4_ electrode after 600 cycles. The tortuosity color scale ranges from 1.0 (blue) to 1.8 (red). The pore network node color represents the equivalent pore radius (0 to 1 um). g-i) SEM images of the bare cathode material g), the cathode material in 0 wt% h), and 0.5 wt% i) g-C_3_N_4_ electrodes after 600 cycles, showing the difference in byproduct accumulation on the cathode material surface. Quantitative parameters are summarized in Table S13.

**Table S14.** Quantitative pore structure parameters obtained from X-ray CT analysis of the 0 wt% and 0.5 wt% g-C_3_N_4_ electrodes before and after 600 charge-discharge cycles. Values in parentheses indicate the relative change from the before-cycling state.

| **Parameter** | **0 wt%**  **(before)** | **0 wt%**  **(After 600 cycle))** | **0.5 wt%**  **(before)** | **0.5 wt%**  **(After 600 cycle))** |
| --- | --- | --- | --- | --- |
| Pore Volume (%) | 32.08 | 29.42 (-8.3%) | 32.46 | 31.26 (-3.7%) |
| Tortuosity | 1.1883 | 1.6535 (+39.1%) | 1.203 | 1.3991 (+16.3%) |
| Coordination Number | 5.301 | 4.363 (-17.7%) | 4.886 | 4.412(-9.7%) |

**Post-mortem XPS analysis of g-C_3_N_4_ structural stability and CEI evolution.**

To assess the long-term structural integrity of the g-C_3_N_4_ additive and its influence on the cathode-electrolyte interphase (CEI), post-mortem XPS analysis was performed on both the 0 wt% and 0.5 wt% g-C_3_N_4_ electrodes after 600 cycles in pouch full-cells.

**1. N 1s analysis (Figure S19a and b)**

The N 1s spectrum of the 0.5 wt% g-C_3_N_4_ electrode after formation cycling (Figure S19a) shows four characteristic peaks: sp^2^ hybridized nitrogen (C=N–C) at 398.5 eV, Li–N coordination at 398.7 eV, bridging nitrogen (N–(C)_3_) at 399.5 eV, and terminal N–H at 400.8 eV. After 600 cycles (Figure S19b), the sp^2^ N and bridging N peaks are clearly preserved at their original binding-energy positions. The retention of these primary structural signatures of the heptazine framework confirms that g-C_3_N_4_ does not undergo severe structural evolution or dissolution during prolonged cycling.

**2. F 1s analysis (Figure S19c and d)**

The 0 wt% electrode (Figure S19c) shows a CEI containing pronounced organic fluorine species, including –CF_2_–/C–F and P–F components, with a relatively smaller LiF contribution. In contrast, the 0.5 wt% g-C_3_N_4_ electrode (Figure S19d) exhibits an enhanced LiF contribution relative to organic fluorine species. This indicates that g-C_3_N_4_ promotes the formation of a more inorganic-rich CEI, which is generally associated with improved chemical and mechanical stability of the interphase.

**3. O 1s analysis (Figure S19e and f)**

The 0 wt% electrode (Figure S19e) is dominated by oxygen-containing organic CEI components, while the metal-oxygen (M–O) lattice signal is largely obscured by the overlying interphase. In the 0.5 wt% g-C_3_N_4_ electrode (Figure S19f), the M–O lattice signal is more clearly recovered, accompanied by reduced organic CEI components. This result is consistent with a thinner and more stable CEI on the g-C_3_N_4_-containing electrode.

Taken together, these post-mortem XPS results show that g-C_3_N_4_ remains structurally persistent over prolonged cycling and modulates the CEI toward a more inorganic-rich and less organic-decomposition-dominated interphase. This interpretation is consistent with the post-mortem SEM and X-ray CT analyses (Figure S18), where the 0.5 wt% g-C_3_N_4_ electrode exhibited a cleaner cathode surface, less pore clogging, and better preservation of pore connectivity after 600 cycles.

**Figure S19.** Post-mortem XPS spectra of cathode electrodes after 600 cycles. N 1s spectra of the 0.5 wt% g-C_3_N_4_ electrode (a) after formation and (b) after 600 cycles, confirming the structural persistence of the g-C_3_N_4_ framework. F 1s spectra of (c) 0 wt% and (d) 0.5 wt% g-C_3_N_4_ electrodes after 600 cycles. O 1s spectra of (e) 0 wt% and (f) 0.5 wt% g-C_3_N_4_ electrodes after 600 cycles.

**Dual layer electrode manufacturing**

To assess the effectiveness of nitrogen in g-C_3_N_4_ in attracting lithium ions, a dual-layer electrode was fabricated using electrodes with 0.0% and 0.5% g-C_3_N_4_ ratios. Electrodes with different ratios were prepared at a current density of ~4.3 mAh cm^-2^. Before attaching the current collector, the two electrodes should be assembled in the following configurations: 0.0% and 0.0% electrodes, 0.0% electrode at the bottom and 0.5% electrode on top, and 0.5% electrode at the bottom and 0.0% electrode on top. After assembling the two electrodes, carbon-coated aluminum was utilized as the current collector to enhance adhesive force and conductivity with the dry electrodes.

**Figure S20.** a-c) Cross-sectional SEM images of dual-layer electrodes fabricated by stacking two dry electrodes, each with a loading of approximately 20 mg/cm². a) Dual-layer electrode composed entirely of g-C₃N₄-free layers, b) electrode with a g-C₃N₄ 0.5 wt% layer placed on top of a g-C₃N₄-free layer, and c) electrode with a g-C₃N₄ 0.5 wt% layer placed beneath a g-C₃N₄-free layer. d) Rate capability performance of the fabricated dual-layer electrodes. e) The discharge voltage profile of dual-layer electrode at 1.0C.

**Table S15.** Physical properties of dual-layer electrodes composed of two stacked dry electrodes. The table lists the loading level, thickness, electrode density, specific capacity, norminal voltage and power density for three configurations: electrodes without g-C₃N₄ in both layers, electrodes with g-C₃N₄ (0.5 wt%) in the top layer only, and electrodes with g-C₃N₄ (0.5 wt%) in the bottom layer only. All electrodes exhibited similar overall thickness (~113 µm), ensuring consistent comparisons of electrochemical performance.

| **Sample** | **Loading level (mg/cm^2^)** | **Thickness**  **(um)** | **Electrode density (g/cc)** | **1C_Specific capacity**  **(mAh/g)** | **Norminal voltage (V)** | **Electrode**  **power density**  **(W/kg)** |
| --- | --- | --- | --- | --- | --- | --- |
| g-C_3_N_4_ 0.0%/0.0% | 42.09 | 113 | 3.58 | 56.9 | 3.57 | 203.1 |
| g-C_3_N_4_ 0.5%/0.0% | 42.86 | 113 | 3.64 | 68.5 | 3.57 | 244.4 |
| g-C_3_N_4_ 0.0%/0.5% | 41.78 | 113 | 3.55 | 89.6 | 3.60 | 322.5 |

**Figure S21.** Quantitative trade-off analysis between Li-N coordination bonding benefits and resistance penalties. a) Second-order polynomial fit of 3C discharge capacity versus g-C_3_N_4_ content. b) Correlation between 3C discharge capacity and electronic resistance, highlighting the resistance-dominated regime above 0.5 wt%. c) Marginal-effect analysis (dC/dx) identifying the crossover point at 1.26 wt%, beyond which the resistance and structural penalties dominate the marginal change in capacity. d) Comparison of capacity improvement and resistance increase relative to the 0 wt% baseline.

**Figure S22.** Mechanistic analysis of the charge transfer resistance (R_ct_) evolution. a) Nyquist plots from EIS measurements at 303.15 K, with the inset table showing the fitted series resistance (R_s_) and charge transfer resistance (R_ct_). b) The non-linear trend of Rct governed by a three-stage dominant mechanism: (Stage 1) Li-N bonding lowers the desolvation energy barrier; (Stage 2) the intrinsic electronic resistance of g-C_3_N_4_ impedes electron transfer; and (Stage 3) spring-back expands pore volume, reactivating Li-N bonding and lowering local R_ct_. c) Correlation between pore volume expansion and reduced pore connectivity (coordination number) at high g-C_3_N_4_ contents. d) The paradox between local interfacial kinetics (R_ct_) and global 3C discharge capacity, highlighting that poor long-range transport restricts overall performance despite fast local reactions.

**Reference**

(1) Ogihara, N.; Kawauchi, S.; Okugawa, T.; Egashira, T.; Kobayashi, Y. J. Electrochem. Soc. 2012, 159, A1034-A1039.

(2) Thorat, I. V.; Stephenson, D. E.; Zacharias, N. A.; Zaghib, K.; Harb, J. N.; Wheeler, D. R. J. Power Sources 2009, 188, 592-600.

(3) Ebner, M.; Geldmacher, F.; Marone, F.; Stampanoni, M.; Wood, V. Adv. Energy Mater. 2014, 4, 1301278.
